# Polycysteine as a new type of radio-protector ameliorated tissue injury through inhibiting ferroptosis in mice

**DOI:** 10.1038/s41419-021-03479-0

**Published:** 2021-02-18

**Authors:** Junling Zhang, Kui Li, Qianru Zhang, Zhimei Zhu, Gongchao Huang, Hongqi Tian

**Affiliations:** 1 Tianjin Key Laboratory of Radiation Medicine and Molecular Nuclear Medicine, Institute of Radiation Medicine, Chinese Academy of Medical Science & Peking Union Medical College, 300000 Tianjin, China; 2 KeChow Pharma, Inc., 201203 Shanghai, China

**Keywords:** Drug screening, Drug development

## Abstract

Amifostine has been the only small molecule radio-protector approved by FDA for decades; however, the serious adverse effects limit its clinical use. To address the toxicity issues and maintain the good potency, a series of modified small polycysteine peptides had been prepared. Among them, compound **5** exhibited the highest radio-protective efficacy, the same as amifostine, but much better safety profile. To confirm the correlation between the radiation-protective efficacy and the DNA binding capability, each of the enantiomers of the polycysteine peptides had been prepared. As a result, the l-configuration compounds had obviously higher efficacy than the corresponding d-configuration enantiomers; among them, compound **5** showed the highest DNA binding capability and radiation-protective efficacy. To our knowledge, this is the first study that has proved their correlations using direct comparison. Further exploration of the mechanism revealed that the ionizing radiation (IR) triggered ferroptosis inhibition by compound **5** could be one of the pathways for the protection effect, which was different from amifostine. In summary, the preliminary result showed that compound **5**, a polycysteine as a new type of radio-protector, had been developed with good efficacy and safety profile. Further study of the compound for potential use is ongoing.

## Introduction

Total body irradiation (TBI) may induce injury in many tissues and organs. Direct action destroys cellular molecular structure, leading to abnormal function and metabolic disorders. Indirect action occurs via the generation of free radicals by water radiolysis^1^, inducing DNA damage and further increase in the cellular free radical level^2,3^. The damage by high-dose ionizing radiation (IR) to biological macromolecules is fatal, which can lead to individual death in a short time, and the attacking on DNA by the free radicals is the main cause of death at low and moderate radiation doses. So free radical scavenging is usually considered as the main method for alleviating TBI-induced injury.

It has been reported that many kinds of anti-oxidative compounds have radiation protective effect^4–6^. However, few of these compounds have clear efficacy and superior performance. Amifostine, a kind of aminothiols, is the only drug approved by FDA for application of xerostomia with radiotherapy of head and neck cancer. Over decades, thiol in amifostine and other aminothiol radiation protectors had been considered as the functional chemical group which can scavenge free radicals and protect against radiation-induced injury effectively. However, it seems that number of thiols in the compound is not the only factor to evaluate the radio-protective efficacy. For example, the molecular weight of *N*-acetyl-cysteine (NAC) is less than that of amifostine, there should be more thiols when irradiated mice were treated with NAC at the same dose as amifostine; however, NAC is much less effective on protecting radiation-induced injury^7^.

Smoluk and colleagues reported that drug–DNA binding capability could be one of the factors for the radioprotection efficacy^3,8^. Amifostine is delivered as a prodrug, and dephosphorylated to active metabolite (WR-1065) by alkaline phosphatases^9^; WR-1065 concentrates near the DNA, providing a sufficiently high local concentration to be effective in scavenging reactive oxygen species (ROS)^3^. It has been reported that the net charge on the thiol is a significant factor in the radiation protective efficacy of the compound^10–12^. Fahl and colleagues reported that the positively charged alkyl-amine backbone binding to the negatively charged DNA backbone provided the most potent radio-protective aminothiol compound^13,14^. To increase the drug–DNA affinity and ionic interaction and so as to improve the radio-protection efficacy, they synthesized a series of polyamine thiol compounds. However, the most effective molecule was Prc-210 with two amino groups, not other more polyamine thiol compounds^14^. Therefore, the clear correlation between the drug–DNA binding capability and the subsequent radiation protective efficacy was not established yet with direct evidence.

In this study, we designed a series of modified polycysteine peptides, including compounds **1**, **2**, **3**, **4**, **5**, **6**, **7**, **8**, and **9**, aimed to keep the radio-protection efficacy same as amifostine, and in the meantime, to improve the safety profile better, and to elicit the correlation between drug–DNA binding capability and the subsequent radiation protective efficacy. There are three important concepts were systematically considered in our compound designing: firstly, the polycysteine peptide backbone to alleviate the toxicity possibly caused from the polyamine, which is the amifostine structure backbone; secondly, the number of terminal thiol segments in the amide side chain backbone is increased to elevate ROS scavenging ability; thirdly, the perpendicular, alkyl sidechain with a terminal thiol projected away from the DNA backbone to enable ROS scavenging around DNA, the same idea as Prc-210. Among the compounds, compound **5** showed the highest DNA binding capability and gave the highest radio-protection efficacy with the dose reduction factor (DRF) value 1.61, which is similar to amifostine with DRF value 1.71. Compound **5** also give the expected better safety profile than amifostine because of the polycysteine peptide backbone. It was worth noting that among these small molecular compounds, the radioprotective efficacy and DNA binding strength of the l-configuration was far more effective than that of the corresponding d-configuration compounds, probably because of the stereo-mismatch between DNA and the d-configuration drug. The result showed a very clear trend: the compounds, with higher drug–DNA binding capability, exhibited higher radiation protection effect. To our knowledge, it is the first time to prove the concept with direct evidence: the higher drug–DNA binding capability, the higher radioprotection efficacy.

Pre-treatment of compound **5** alleviated IR-induced hematopoietic system, lung, and small intestine (SI) injury in mice. Furthermore, the RNA sequence results showed that compound **5** could regulate redox balance, amino acid synthesis, and metabolism pathways in irradiated mice SI crypt. As we know, these pathways are tightly linked to the regulation of ferroptosis. Ferroptosis is a form of non-apoptotic cell death driven by lipid peroxidation, which is rarely concerned and illustrated in IR-induced SI injury. We demonstrated that IR induces lipid peroxidation and ferroptosis in SI, compound **5** can inhibit IR and some ferroptosis activator-triggered ferroptosis. These results demonstrated that the ferroptosis mechanism might be involved in the radiation protection for the polycysteine as the new type of radioprotector.

## Materials and methods

### Synthetic scheme for compound 5

**Fig. 1:**
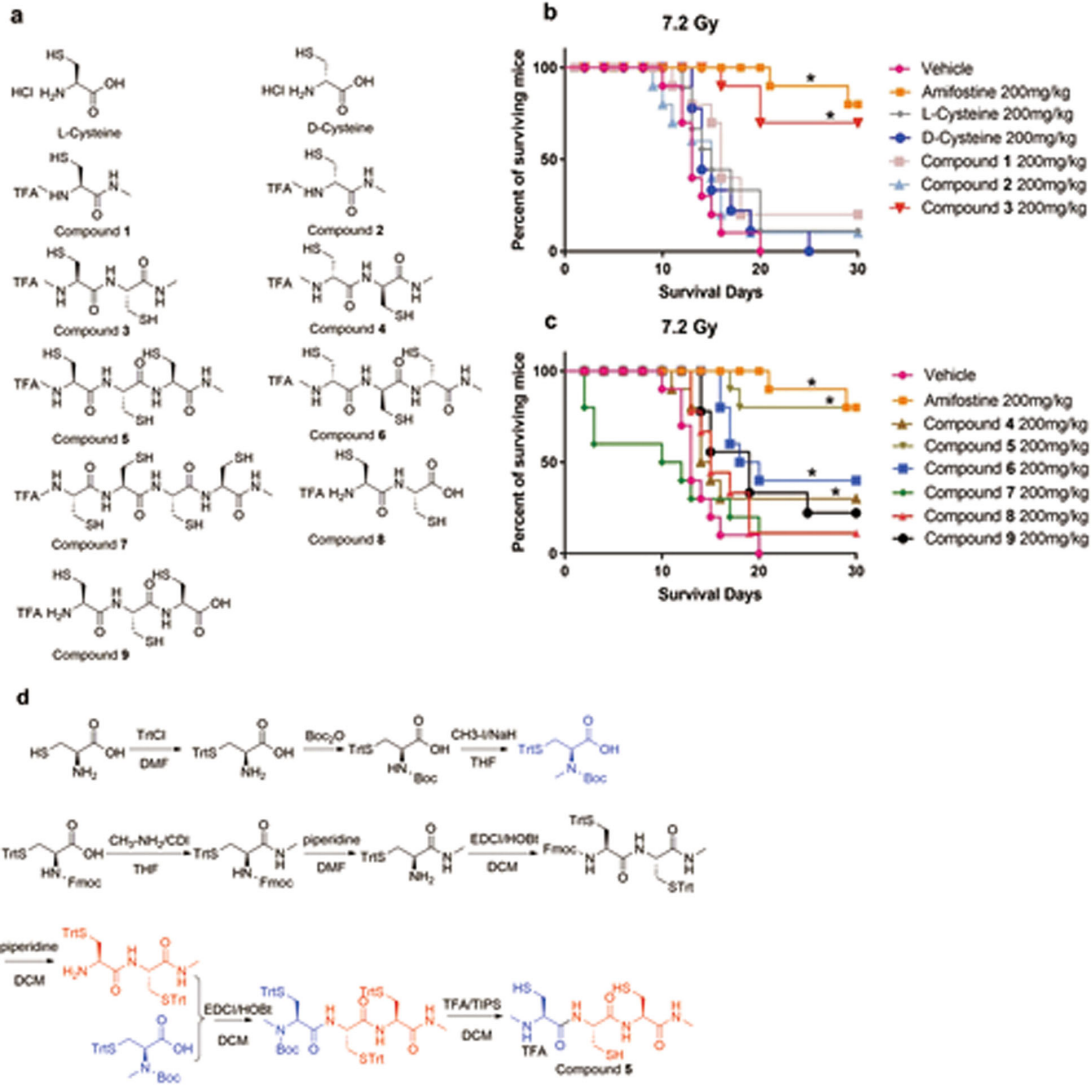
Chemical structure of compounds and their 30-days survival rate after exposed to lethal TBI in mice. **a** Chemical structure of cysteine, derivatives, and new compounds **1–9**; **b** Survival rate of irradiated mice treated with l-cysteine, d-cysteine, and compounds **1–3**. **c** Survival rate of irradiated mice treated with compounds **4–9**; **d** Synthesis scheme of compound **5**; *p < 0.05 vs 7.2 Gy, *N* = 10 in each group.

The synthesis of compound **5** was started from l-cysteine, the free -SH and –N-H_2_ of which was protected by TrtCl and Boc_2_O successively affording *N*-(tert-butoxyca-rbonyl)-*S*-trityl-l-cysteine, then the protected *N*-(tert-butoxycarbonyl)-*N*-methyl-*S*-trityl-l-cysteine was obtained by methylation of *N*-(tert-butoxycarbonyl)-*S*-trityl-l-cysteine. After that, the *N*-(tert-butoxycarbonyl)-*N*-methyl-*S*-trityl-l-cysteine was condensed with (R)-2-amino-*N*-((R)-1-(methylamino)-1-oxo-3-(tritylthio) propan-2-yl)-3-(tritylthio) propan-amide ((R)-2-amino-*N*-((R)-1-(methylamino)-1-oxo-3-(tritylthio) propan-2-yl)-3-(tritylthio)-propanamide was prepared from the commercially available *N*-(((9H-fluoren-9-yl) meth-oxy) carbonyl)-*S*-trityl-l-cysteine through four steps reaction) to afford the tert-butyl meth-yl ((4R, 7R, 10R)-3,6,9-trioxo-13,13,13-triphenyl-4,7-bis((tritylthio) methyl)-12-thia-2,5,8-tr-iazatridecan-10-yl) carbamate. Finally, the target molecular compound 5 was obtained with total yield of 7.4% by treating tert-butyl methyl ((4R,7R,10R)-3,6,9-trioxo-13,13,13-triphenyl-4,7-bis((tritylthio)methyl)-12-thia-2,5,8-triazatridecan-10-yl) carbamate with TFA and TIP-S. Synthesis scheme of compound **5** was shown in Fig. 1d.

### Evaluation of DNA binding with UV–spectroscopy study

To evaluate the interaction between compounds and DNA, the UV-spectroscopy were recorded on a Shimadzu UV-1750 spectrophotometer. The absorbance values of calf-thymus DNA (CT DNA) (100 ng/μL) and CT DNA–compounds complex were detected in the wavelength range of 260–330 nm; the solution of compounds at concentrations of 0–30 mM was prepared in DMSO-phosphate buffer (PH = 7.4). The double reciprocal plot of 1/(*A*−*A*_0_) vs 1/(compound concentration) is linear and the binding constant (*K*) of compounds–CT DNA adducts was calculated according to the method described by Connors^15^.

$$K = \frac{{{\mathrm{Intercept}}}}{{{\mathrm{Slope}}}}$$

### Mice

Male C57BL/6 mice and BALB/c-Nude mice were purchased from Beijing Huafukang Bioscience Co. Inc (Beijing, China). Mice in survival experiment, hematopoietic system, and SI experiments were used at approximately 8–10 weeks of age with a body weight of 21–22 g. Mice in lung fibrosis experiment were used with a body weight more than 25 g. All of the animal experiments in our study were approved by the Animal Care and Ethics Committee at the Institute of Radiation Medicine. The study was performed in accordance with the principle of Institutional Animal Care and Ethics Committee guidelines.

### Radiation and compound 5 administration

C57BL/6 mice were randomly divided into three groups in survival experiment, radiation, radiation+amifostine, and radiation+compound **5**; for other experiments, mice were divided into six groups, control, control+amifostine, control+compound **5**, radiation, radiation+amifostine, and radiation+compound **5**. The mice were exposed to γ-ray (4 Gy, 7.2 Gy, 7.5 Gy, 10 Gy, 12.5 Gy) whole body irradiation (WBI) at a dose rate of 0.99 Gy/min (Gammacell-40 ^137^Cs irradiator, Atomic Energy of Canada Ltd.). Control mice were sham-irradiated. For the abdominal irradiation (ABI) study, the mice were exposed to γ-ray (15 Gy, 18 Gy) ABI at a dose rate of 0.99 Gy/min (Gammacell-40 ^137^Cs irradiator, Atomic Energy of Canada Ltd.). Control mice were sham-irradiated. IR was delivered using a lead shielding, except for the 2.75 cm diameter roundness exposed field in mouse abdomen and the other parts of the mouse were shielded. For lung fibrosis, experiments were exposed to 17 Gy X-rays at a dose rate of 3.23 Gy/min using RS2000 irradiator (Rad Source Technologies, Suwanee, GA, USA). Control mice and mice with administration of compound **5** or amifostine alone were sham-irradiated. For compound **5**, amifostine, liproxstatin-1, and imidazole ketone erastin (IKE) administration, mice were administered intraperitoneally (i.p.) 20 min before TBI; and in liproxstatin-1 and IKE groups, mice will be treated with these compounds for another 4 days after TBI. Control mice and TBI mice received the same volume of vehicle (normal saline, NS) at the same time point.

### Peripheral blood cell counts and hematopoietic stem/progenitor cells analysis

Blood was obtained from mice via the orbital sinus and was collected into micro-pipettes coated with EDTA. *K*_3_, white blood cell (WBC) counts, red blood cell (RBC) counts, hemoglobin (HGB), platelet (PLT), the percentage of neutrophil granulocytes (NE%), and the percentage of lymphocytes (LY%) were calculated on a hematology analyzer (Nihon kohden, Japan). The bone marrow cells (BMCs) number was also detected using the above analyzer. For the analysis of hematopoietic stem/progenitor cells (HSPCs), 5 × 10^6^ BM cells were obtained from the mice femur. The cells were first incubated with the lineage mix cocktail (Ter119, Gr1 (clone RB6-8C5, Biolegend, San Diego, CA, USA), CD11b (clone M1/70, Biolegend, San Diego, CA, USA), B220 (clone RA3-6B2, Biolegend, San Diego, CA, USA), CD4 (clone GK1.5, Biolegend, San Diego, CA, USA), and CD8 (clone 53-6.7, Biolegend, San Diego, CA, USA)) for 30 min at 4 °C, and then incubated with streptivadin-Percp (clone145-2C11, Biolegend, San Diego, CA, USA), Sca1-PE (clone D7, eBioscience, San Diego, CA, USA), c-kit-APC (clone 2B8, eBioscience, San Diego, CA, USA), and CD34-FITC (clone RAM34, eBioscience, San Diego, CA, USA) for 90 min at 4 °C. Data were collected using a BD Accuri C6 flow cytometer (BD Bioscience, San Jose, CA, USA).

### Histological analyses, Masson staining in lung tissue

Lobe inferior in the right lung was obtained immediately after the mice were euthanized, then it was fixed in 10% formalin for at least 24 h. The lung tissue was dehydrated through graded alcohols and xylene, embedded in paraffin, and coronally sectioned at a thickness of 5 μm. Hematoxylin and eosin (H&E) staining was performed at 2nd, 4th, and 6th month after the mice were exposed to lung radiation following the standard procedure, including de-paraffinized, re-hydrated, stained with H&E. For evaluating the collagen deposition, sections from paraffin-embedded samples were de-paraffinized and processed for a Masson trichrome staining according to the manufacturer’s instructions (Solarbio, Beijing, China).

### Histological analyses, TUNEL, and 5-bromo-2ʹ-deoxyuridine staining in SI

The middle section of jejunum was obtained immediately after the mice were euthanized; the jejunum (about 10 cm in length) was rinsed with ice-cold PBS, and then it was fixed in 10% formalin for at least 24 h. H&E staining was performed following the above description. Relative height of villi was calculated by an Image-Pro Plus software (Version 6.1; Media Cybernetics). Analysis of apoptosis was performed using terminal deoxynucleotidyl transferase-mediated dUTP-biotin nick end labeling (TUNEL) assay (Roche, Basel, Switzerland) according to the manufacturer’s instructions. For 5-bromo-2ʹ-deoxyuridine (BrdU) staining, mice were injected with 100 mg/kg BrdU (Sigma, St. Louis, MO, USA) 2 h prior to sacrifice, sections from jejunum were deparaffinized and then a Brdu immuno-histochemistry kit was used according to the manufacturer’s instructions (Abcam, Cambridge, UK, USA) with modification, which epitope retrieved was executed 1 min by high pressure in a mix of citric acid and citrate solution. Apoptosis and proliferation were evaluated by TUNEL and Brdu-positive cells per crypt. All the images were captured using a Leica DMi8 fluorescence microscope (Leica, Solms, Germany).

### RNA-sequence in SI crypt cells

Crypt cells were isolated from SI as previously reported^16,17^. In brief, after the mice were euthanized, SI was collected immediately, and the organ was opened longitudinally and washed with PBS carefully. The SI was cut into pieces with a length of about 0.2 cm. To dissociate the crypts, the SI was incubated at 4 °C in EDTA (10 mM) for 15 min and then in EDTA (5 mM) for an additional 15 min. After neutralizing the enzyme activity by fetal bovine serum (FBS), crypts were collected and stored in 1 mL Trizol. RNA was isolated, and a total amount of 3 μg of RNA was used as input material. Sequencing libraries were generated using NEBNext® UltraTM RNA Library Prep Kit for Illumina® (NEB, USA) following the manufacturer’s recommendations, and index codes were added to attribute sequences to each sample. The clustering of the index-coded samples was performed on a cBot Cluster Generation System using TruSeq PE Cluster Kit v3-cBot-HS according to the manufacturer’s instructions. After cluster generation, the library preparations were sequenced on an Illumina Hiseq platform and 125 bp/150 bp paired-end reads were generated. Differential expression analysis (three biological replicates per group) was performed using the DESeq2 R package (1.10.1). We used cluster Profiler R package to test the statistical enrichment of differential expression genes in reactome pathways.

### Quantitative real-time PCR

Total RNA was isolated from the SI crypt and then reversed to cDNA using the Revert Aid First Strand cDNA Synthesis Kit (Thermo Scientific, Waltham, MA, USA) follow the manufacture’s instructions. All the sequences of the primers were listed in Supplementary Table 2. PCR experiments were conducted using an ABI 7500 Sequence Detection System (Life Technologies, Grand Island, NY, USA).

### Cell culture and irradiation

Rat intestinal epithelioid cell line IEC6 was obtained from Enzyme Research Biotechnology Co., Ltd (Shanghai, China), and cultured in Dulbecco’s Modified Eagle Medium (DMEM) (Gibco, NY, USA) with 10% FBS (Gibco, NY, USA), penicillin (100 U/mL), streptomycin (100 μg/mL), and incubated at 37 °C in a humidified 5% CO_2_ atmosphere. Cells were treated with 8 Gy (colony-forming assay) or 10 Gy γ-ray (other experiments) at a dose rate of 0.99 Gy/min (Gammacell-40 ^137^Cs irradiator, Atomic Energy of Canada Ltd.). The plates were returned to the incubator for further incubated at indicated times. Then cells were harvested by trypsinizing with 0.25% trypsin (0.25% in phosphate-buffered saline).

### Cell viability and colony-forming assay

Cell viability was assayed using MTT assays (Sigma Chemicals Co., St Louis, MO, USA), according to the manufacturer’s protocol. Exponentially growing cells (1 × 10^4^) were seeded in 96-well plates and allowed to adhere overnight. The next day, cells were treated with CIL 56 (10 μM), erastin (10 μM), or IKE (10 μM). After incubation for 4 h, a dilution series of compound **5** (100 μM (the medium was pretreatment with DTT (100 nM)) were added for next 24 h incubation. DTT was used to stabilize compound **5** activity. To evaluate the protective effect of compound **5** against IR-induced ferroptosis, various concentrations of compound **5**, liproxstatin-1 (50 nM) were diluted in DMSO and were added to each test well respectively. Cells were treated with γ-ray and incubated for next 24 h. The optical density (OD) was read at a wavelength of 490 nm using a Microplate Reader (Thermo Waltham, MA, USA). For the colony-forming experiment, one-thousand IEC6 cells were seeded into a 6-well plate, the cells were cultured for 10 days after exposure to radiation, and then the cells were fixed with paraformaldehyde and stained with Giemsa.

### Glutathione detection

Glutathione (GSH) level in IEC6 cells was measured using the GSH-Glo^TM^ Glutathione Assay follow the manufacturer’s protocol. In brief, 1 × 10^4^ cells were incubated with reaction buffer (mixed with Luciferin-NT substrate and Glutathione S-Transferase) for 30 min at room temperature (RT), and then incubated with lyophilized detection buffer for 15 min at RT; the luminescence value was detected using the microplate Reader. GSH level was expressed as the ratio fold to the 10 Gy.

### Analysis of ROS level in IEC6 cells

Total ROS was detected with 2, 7-dichlorodihydrofluorescein diacetate (DCFH-DA, Beyotime Biotechnology, Nanjing, China; 10 μΜ) for 20 min; lipid ROS was detected with C11-Bodipy (ThermoFisher Scientific, Waltham, MA, USA; 2 μΜ) for 30 min; mitochondrial ROS was detected with MitoSox (ThermoFisher Scientific, Waltham, MA, USA; 10 μΜ) for 15 min, and superoxide free radicals were detected with dihydroethidium (DHE, Beyotime Biotechnology, Nanjing, China; 5 μM) for 10 min at 37 °C in an incubator. The BD Acurri C6 flow cytometer (BD Bioscience, San Jose, CA, USA) was used to detect the fluorescence intensity (MFI); ROS level was expressed as the ratio fold to the control.

### Western blotting

Crypt cells were isolated from SI as described above, IEC-6 cells were collected and the total proteins were extracted at 24 h after irradiation of 10 Gy. Cells were lysed in ice-cold RIPA buffer with 1.0 mM protease inhibitor and the total protein was quantified using the bicinchoninic acid (BCA) protein assay kit. For western blotting analysis, equal amounts of proteins were separated by 10% sodium dodecyl sulfate-polyacrylamide gel electrophoresis (SDS-PAGE), followed by transfer onto a nylon membrane (Merck Millipore Ltd); the membranes were blocked with blocking buffer (0.1% Tween 20 in 0.2 M Tris–base and 1.5 M NaCl aqueous solution, 5% skimmed milk). Subsequently, the membranes were incubated with the primary antibodies GPX-4 (1:5000 dilution), NOX1 (1:500), and β-actin (1:5000 dilution). This was followed by incubation with suitable HRP-conjugated secondary antibody. The protein bands were visualized using ECL chemiluminescence reagents. The intensities of each protein band were measured using Image Lab^TM^ software (Bio-Rad, USA).

### Statistical analysis

A Mann–Whitney *U* test was used for the majority of comparisons. Data in survival experiment were analyzed for statistical significance using *long-rank* test. Statistical analyses were performed using GraphPad Prism 8 software. A *p* < 0.05 represented statistical significance.

## Results

### Chemical synthesis of a series of compounds and their radiation protective effect on mice

As shown in Fig. 1a, a total of 11 compounds were used in our experiment to explore their protective effect against radiation-induced injury. Among them, the l-configuration compound **5** has three thiol groups and three amide groups, compound **3** has two thiol groups and two amide groups, compound **7** has four thiol groups and four amide groups, compound **1** has one thiol group and one amide group; the *R*-configuration compound **4**, compound **6**, and compound **2** have two, three, one thiol groups and two, three, one amide groups, respectively. Compound **8** is the dimer of l-cysteine and compound **9** is the trimer of l-cysteine. The synthesis schemes of compounds **1**, **2**, **3**, **4**, **6**, **7**, **8**, and **9** were shown in Supplementary synthetic procedures section.

Then, we executed the 30-day survival experiment, as shown in Fig. 1b, c; when mice were exposed to 7.2 Gy TBI, the survival rate is 0%; 80% mice survived in compound **5** and amifostine-treated mice groups, followed by 70% in compound **3**, 40% in compound **6**, 30% in compound **4**, 20% in compound **9** and compound **1**, 10% in compound **8**, compound **2**, and l-cysteine-treated mice groups. There were no mice survived in compound **7** and d-cysteine-treated mice groups. Our result shows that a series of compounds and cysteine with different configuration had diverse radiation protective effects.

### Binding capability of compounds–DNA adducts by UV spectroscopy

**Fig. 2:**
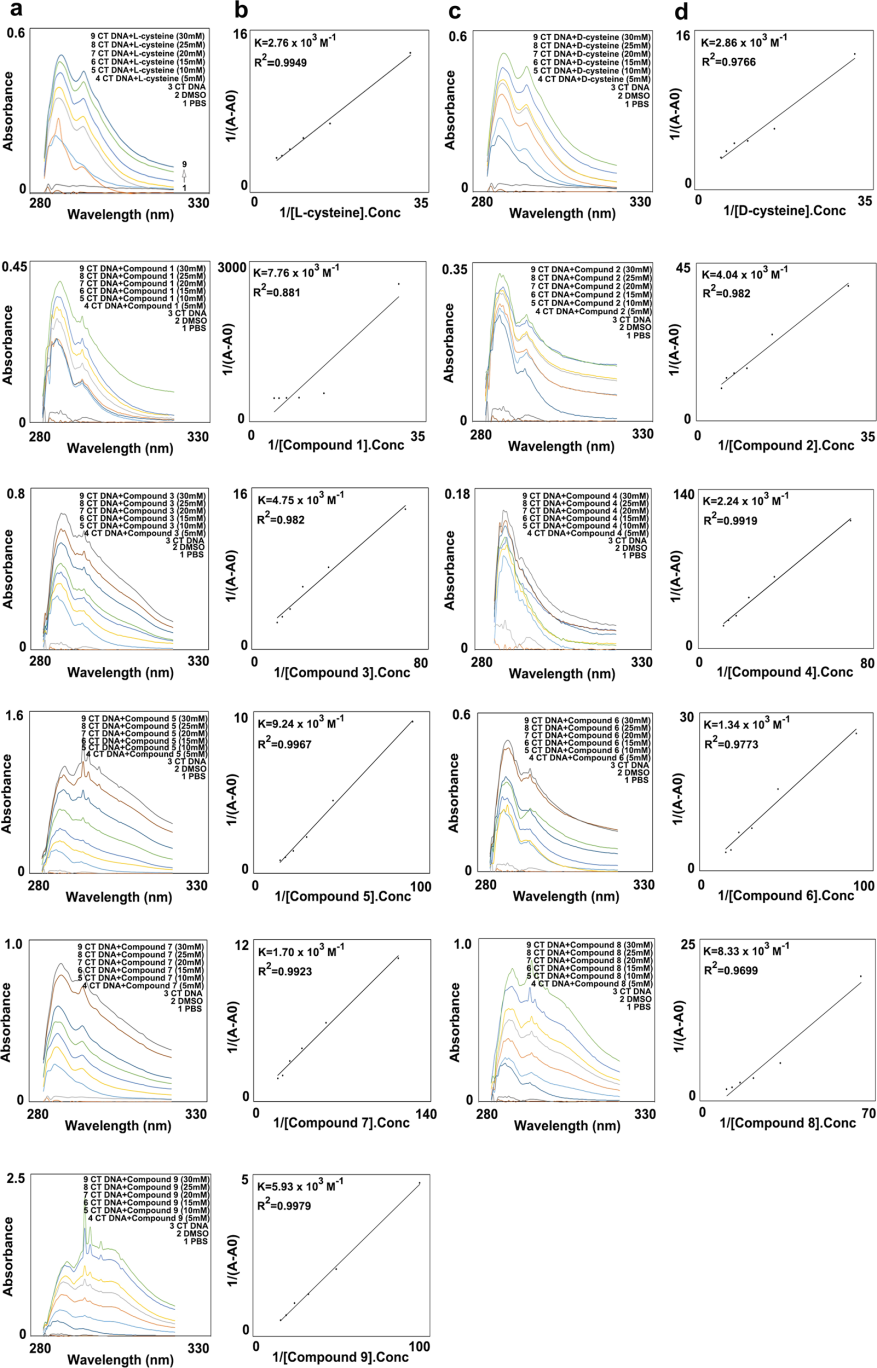
Binding capability of calf-thymus DNA and compounds. **a**, **c** Absorption spectra of CT DNA in the absence and presence of increasing concentrations of small molecular compounds, 5, 10, 15, 20, 25, 30 mM in DMSO-phosphate buffer (PH = 7.4) solution. **b**, **d** Plot of 1/(*A*−*A*_0_) vs 1/[small molecular compounds]. Conc for calculation of binding constant (*K*) of complexes.

UV-spectroscopy is one of the most effective techniques in studying the primal interaction of drug–DNA and complex formation. Figure 2 shows the absorption spectra of CT DNA in the absence and presence of varying concentrations (0 mM–30 mM) of compounds. The double reciprocal plot of 1/(*A*−*A*_0_) vs 1/(compound concentration) is linear and the binding constant (*K*) can be estimated from the ratio of the intercept to the slope, where *A*_0_ is the initial absorbance of the free DNA in the absence of the compound, *A* is the recorded absorbance of complexes at different compound concentrations^18^. The calculated binding constants are *K*_l-cysteine-CT DNA_ = 2.76 × 10^3^ M^−1^; *K*_d-cysteine-CT DNA_ = 2.86 × 10^3^ M^−1^; *K*_compound **1**-CT DNA_ = 7.76 × 10^3^ M^−1^; *K*_compound **2**-CT DNA_ = 4.04 × 10^3^ M^−1^; *K*_compound **3**-CT DNA_ = 4.75 × 10^3^M^−1^; *K*_compound **4**-CT DNA_ = 2.24 × 10^3^ M^−1^; *K*_compound **5**-CT DNA_ = 9.24 × 10^3^M^−1^; *K*_compound **6**-CT DNA_ = 1.34 × 10^3^ M^−1^; *K*_compound **7**-CT DNA_ = 1.70 × 10^3^ M^−1^; *K*_compound **8**-CT DNA_ = 8.33 × 10^3^ M^−1^; *K*_compound **9**-CT DNA_ = 5.93 × 10^3^ M^−1^. The results indicated that the compound with the highest binding capability was compound **5**.

### Compound 5 elevated the survival rate of lethal irradiated mice

**Fig. 3:**
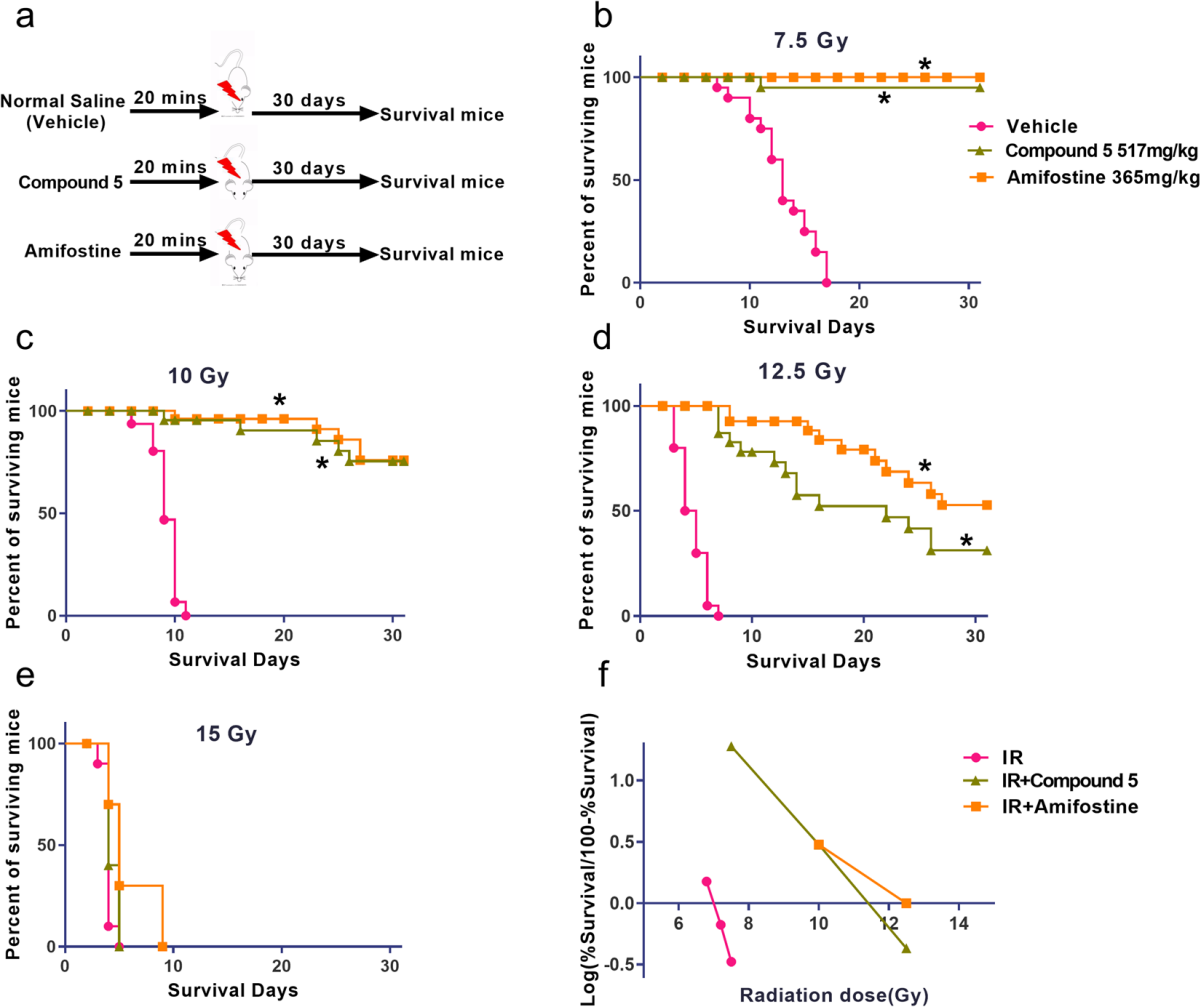
Compound 5 elevated 30-days survival rate of mice exposed to lethal TBI. Mice were administered with normal saline (NS), compound **5**, and amifostine 20 min before exposure to TBI as described in the “Materials and methods” section; control mice were sham-irradiated. **a** Diagram shows the experimental design; survival rate of mice exposed to **b** 7.5 Gy TBI, **c** 10 Gy TBI, **d** 12.5 Gy TBI, and **e** 15 Gy TBI; **f** evaluation of DRF for compound **5** and amifostine; *p < 0.05 vs radiation, *N* = 20 in **b**–**d**, *N* = 20 in **e**.

C57BL/6 mice were exposed to 7.5, 10, 12.5, and 15 Gy TBI. According to the published research^19^, compound **5** is administered at 1/2 maximum tolerance dose (MTD), and MTD is approximately equal to the dose of LD10 dose^19,20^, so in our experiment, 1/2 MTD dose of compound **5** is 517 mg/kg. According to clinical experience, the optimum dose of amifostine in mice is 365 mg/kg. Compound **5** and amifostine were given to mice by i.p. injection 20 min before TBI, then the survival rate was calculated within 30 days after the mice were exposed to TBI. As shown in Fig. 3, 30 days’ survival rate was 0% in all vehicle-treated mice at 7.5 Gy, 10 Gy, 12.5 Gy, and 15 Gy, there were 95%, 75%, 30%, and 0% mice alive after compound **5** treatment at 7.5 Gy, 10 Gy, 12.5 Gy, and 15 Gy group, respectively, and 100%, 75%, 50%, 0% mice alive respectively in amifostine-treated irradiated mice. These data suggest that compound **5** reduces the mortality of irradiated mice significantly even when the dose is up to 12.5 Gy.

### Compound **5** alleviated TBI-induced hematopoietic system injury in mice

**Fig. 4:**
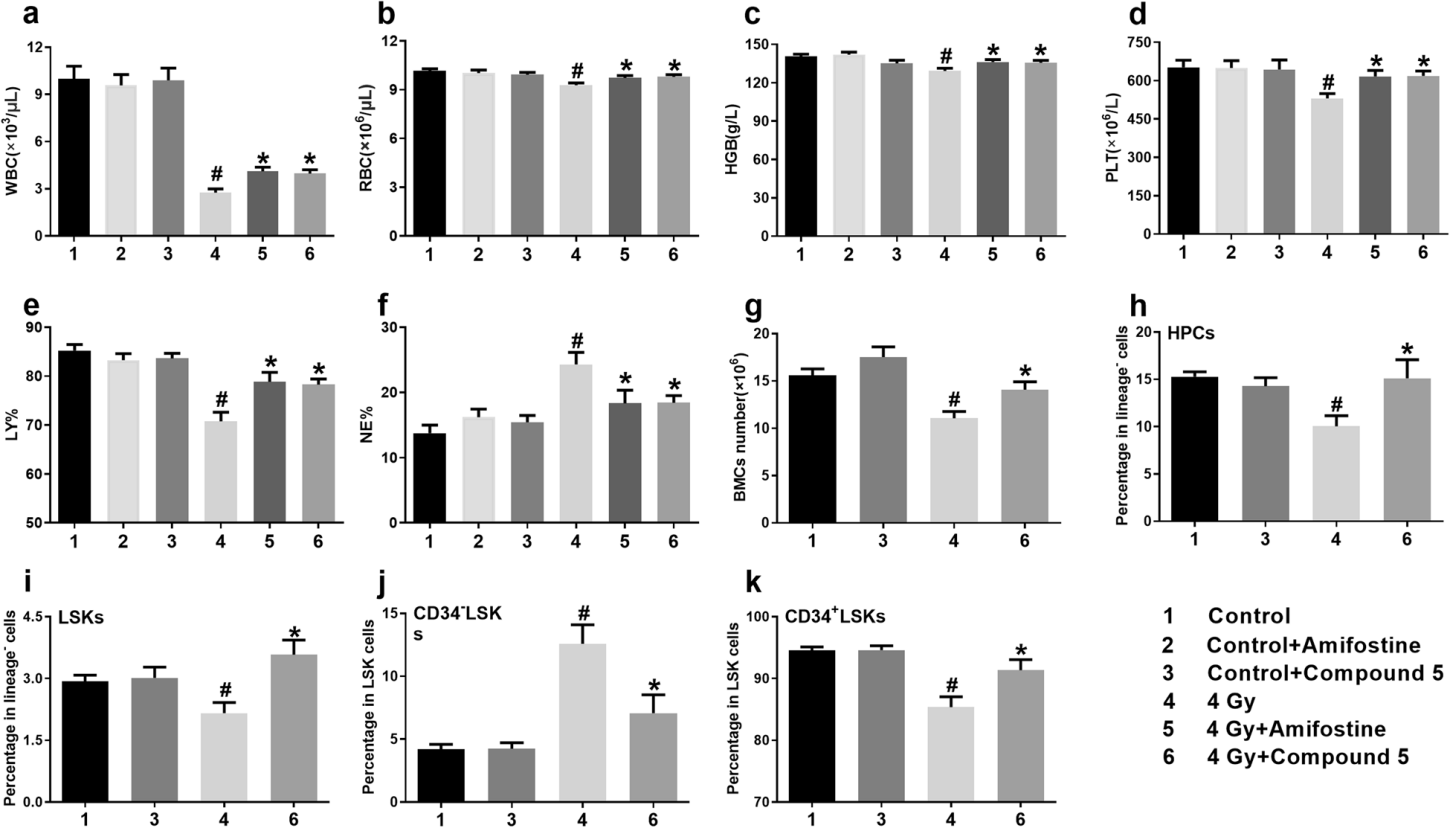
Compound 5 alleviated TBI-induced hematopoietic system injury. Mice were administered with NS, compound **5**, and amifostine 20 min before exposure to 4 Gy TBI as described in the “Materials and methods” section; control mice were sham-irradiated. **a** White blood cells (WBC) count; **b** red blood cells (RBC) count; **c** hemoglobin (HGB) count; **d** platelet (PLT) count; **e** lymphocyte (LY) percentage; **f** neutrophil (NE) percentage; **g** BMCs number; **h** HPCs percentage; **i** LSKs percentage; **j** CD34^−^ LSKs percentage; **k** CD34^+^ LSKs percentage; #*p* < 0.05 vs Control, *p < 0.05 vs 4 Gy, *N* = 25 in group **a**–**f**, *N* = 5 in group **g**–**k**.

C57BL/6 mice were exposed to 4 Gy TBI; 517 mg/kg compound **5** and 365 mg/kg amifostine were given to mice by i.p. injection 20 min before TBI, and then the mice were euthanized at 15th day after TBI. We measured the peripheral blood cell counts with a hematology analyzer to evaluate the differentiation ability of hematopoietic stem cells. As shown in Fig. 4a–f, compared to control group, there is significant decrease in WBC, RBC, HGB, PLT counts, and LY%, and an increase in NE% in mice exposed to 4 Gy TBI; compound **5** increased the counts of WBC, RBC, HGB, PLT, and LY%, and decreased the NE% in 4 Gy TBI irradiated mice. We further detected the BMCs number and HPSCs frequency; as shown in Fig. 4g–k, compared to control group, there was decreased BMCs number, hematopoietic progenitor cells (HPCs) frequency, Lineage^−^Sca1^+^c-kit^+^ (LSKs) frequency, CD34^+^ LSKs frequency, and increased CD34^−^ LSKs frequency; compound **5** could rescue the injury in BMCs number and HPSCs frequency. There was a decrease in spleen and thymus indexes, and an increased ratio of micronuclei in polychromatic erythrocytes. Compound **5** could improve the above hematopoietic system injury effectively (Supplementary Fig. 1). These above data indicate that compound **5** is highly potent to TBI-induced hematopoietic system injury.

### Compound **5** alleviated radiation-induced lung injury

**Fig. 5:**
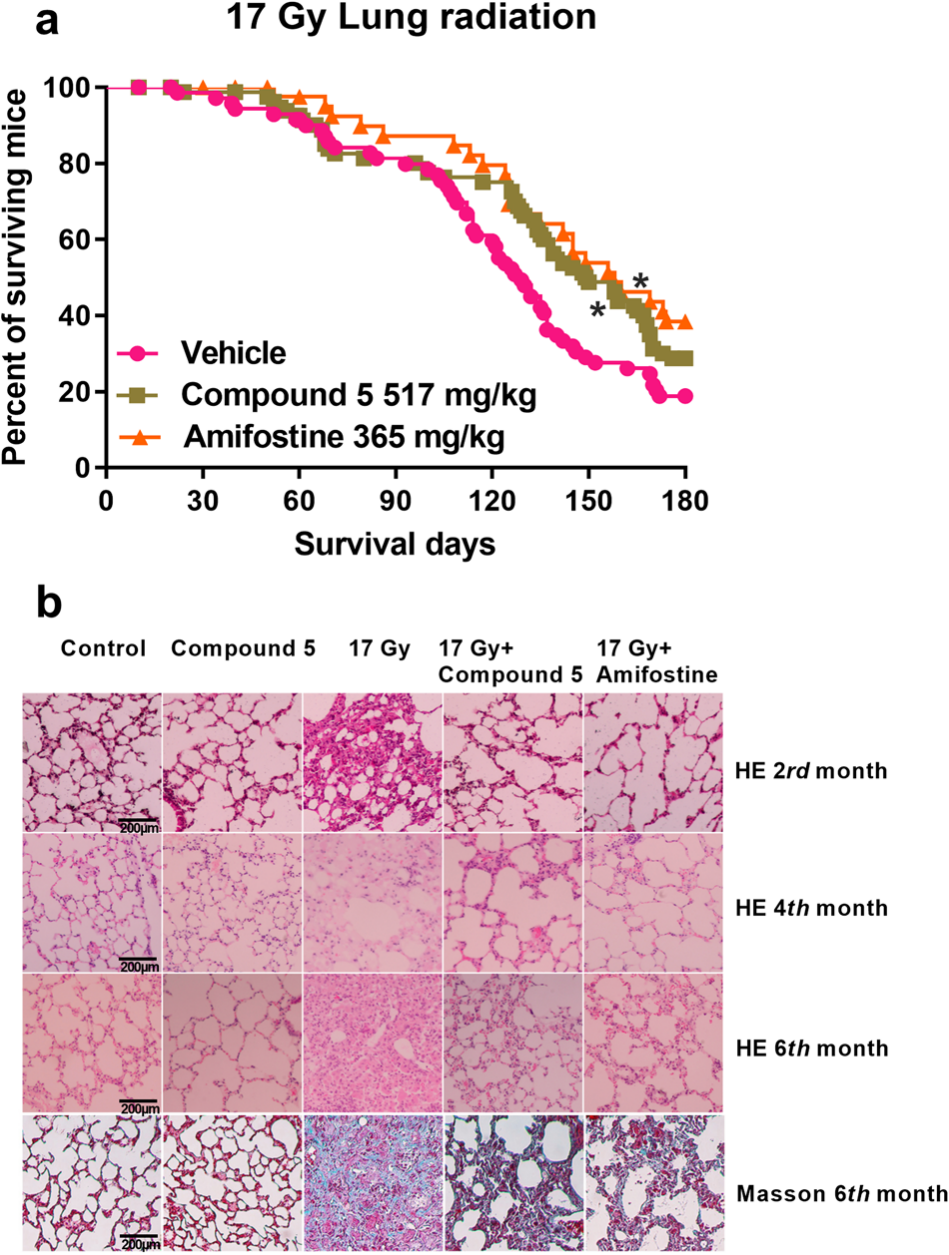
Compound 5 alleviated radiation-induced lung injury. Mice were administered with NS, compound **5**, and amifostine 20 min before exposure to 17 Gy lung radiation as described in the “Materials and methods” section. **a** Survival rate of mice exposed to 17 Gy lung irradiation; **b** representation images of HE and Masson trichrome staining show the lung tissue sections; *p < 0.05 vs 17 Gy, *N* = 69 in vehicle group, *N* = 80 in compound **5** group, *N* = 39 in amifostine group.

C57BL/6 mice were exposed to 17 Gy right lung radiation, and compound **5** or amifostine was injected into mice i.p. 20 min before radiation. The survival rate was calculated within 180 days after radiation; mice were also euthanatized at 8th, 16th, and 24th weeks after radiation to evaluate the degree of fibrosis. As shown in Fig. 5a, there are 18.57%, 28.75%, and 38.46% alive mice in radiation, compound **5**, and amifostine-treated group, respectively, indicating that compound **5** reduced the mortality of mice which were exposed to lung irradiation. The irradiated lung exhibited a persistent alveolar septum width, fibrous exudates, inflammatory cell infiltration, and a strong increase in interstitial collagen fiber deposition as assessed by Masson staining. Treatment of irradiated mice with compound **5** or amifostine significantly reduced the degree of pulmonary fibrosis at all tested time points (Fig. 5b). We recorded the photo of the entire fresh lung 24 weeks after irradiation; there is 66.7% abnormal lung in the vehicle-treated irradiated mice, 40% and 37.5% abnormal lung in the compound **5** and amifostine-treated irradiated mice, respectively (Supplementary Fig. 3). These data suggested that compound **5** could alleviate radiation-induced lung injury.

### Compound **5** alleviated TBI-induced gastrointestinal (GI) syndrome

**Fig. 6:**
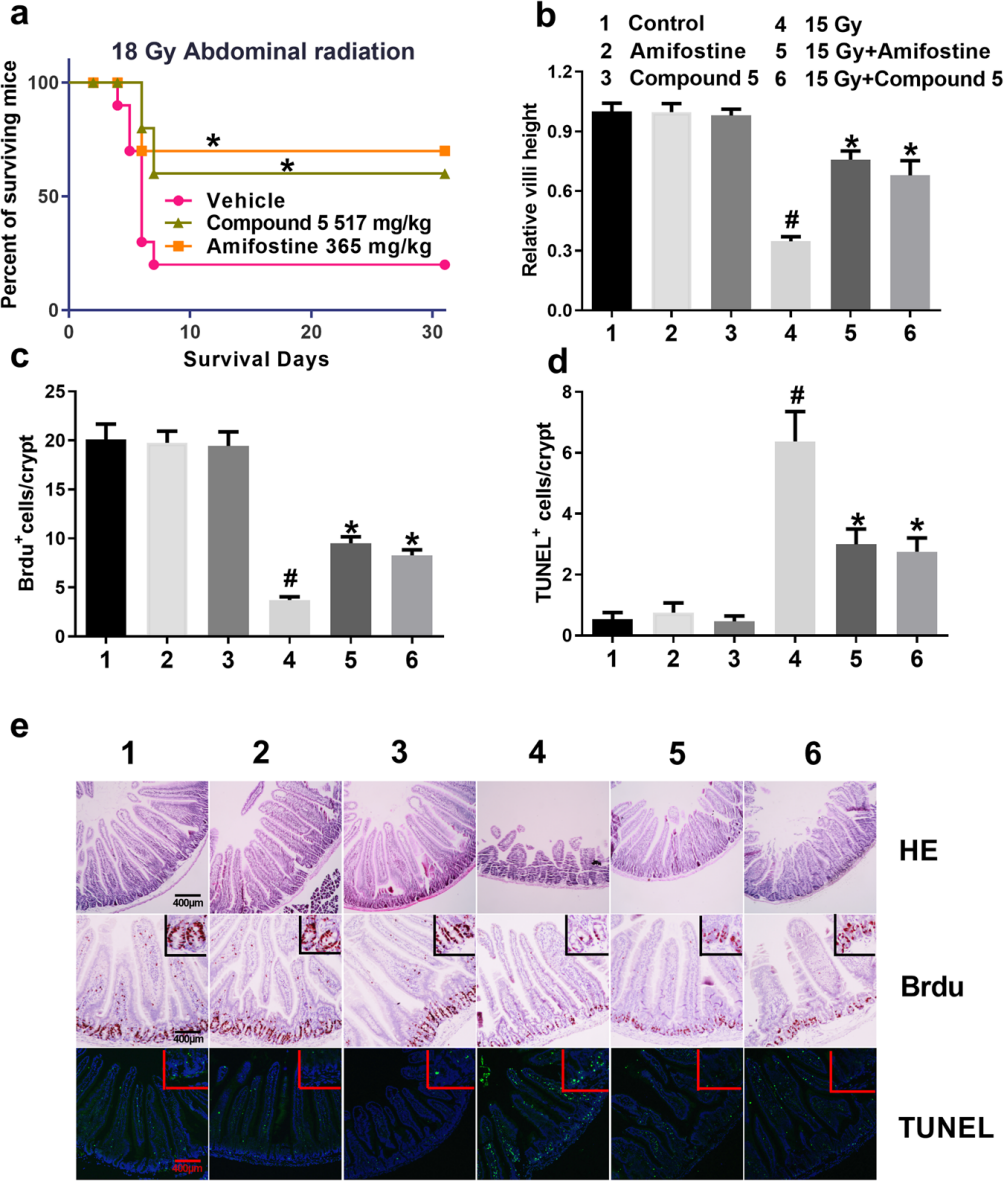
Compound 5 alleviated radiation-induced gastrointestinal (GI) syndrome. Mice were administered with NS, compound **5**, and amifostine 20 min before 15 Gy and 18 Gy ABI as described in the “Materials and methods” section; control mice were sham-irradiated. **a** Survival rate of mice exposed to 18 Gy ABI; **b** bar graph shows the relative villi height in mouse intestine; **c** bar graph shows the percentage of Brdu-positive cells in crypt; **d** bar graph shows the percentage of apoptotic cells in crypt. **e** Representation images of HE, Brdu, and Tunnel staining in mouse intestine sections; #*p* < 0.05 vs Control, *p < 0.05 vs 18 or 15 Gy, *N* = 8–16 in each group.

Mice will die between 7 and 12 days after exposure to radiation at the dose more than 14 Gy, due to SI damage and complications known as GI syndrome^21,22^. To evaluate the radio protective effect of compound **5** on GI syndrome, we first executed a survival experiment; compound **5** and amifostine were injected into mice i.p. 20 min before the mice were exposed to 18 Gy ABI. As shown in Fig. 6a, there were 20% mice alive in vehicle-treated group at 18 Gy, and 60 and 70% in compound **5** and amifostine-treated groups, respectively; there was no significant difference between compound **5** and amifostine-treated mice.

Then mice were exposed to 15 Gy ABI to evaluate the morphology, proliferation, and apoptosis in SI. As shown in Fig. 6b, 5 days after radiation, reduction in relative height of SI villi was ameliorated by compound **5** significantly. Brdu was used to evaluate proliferative cells in SI; compound **5**-treated mice retained the proliferative cells significantly (Fig. 6c). Treatment of mice with compound **5** before 15 Gy ABI reduced the ratio of apoptotic cells (Tunel-positive cells) both in crypt and villus 4 h after radiation (Fig. 6d). The above data indicate that compound **5** alleviated ABI-induced gastrointestinal (GI) syndrome.

### Underlying mechanisms of compound **5** alleviate ABI-induced GI syndrome

**Fig. 7:**
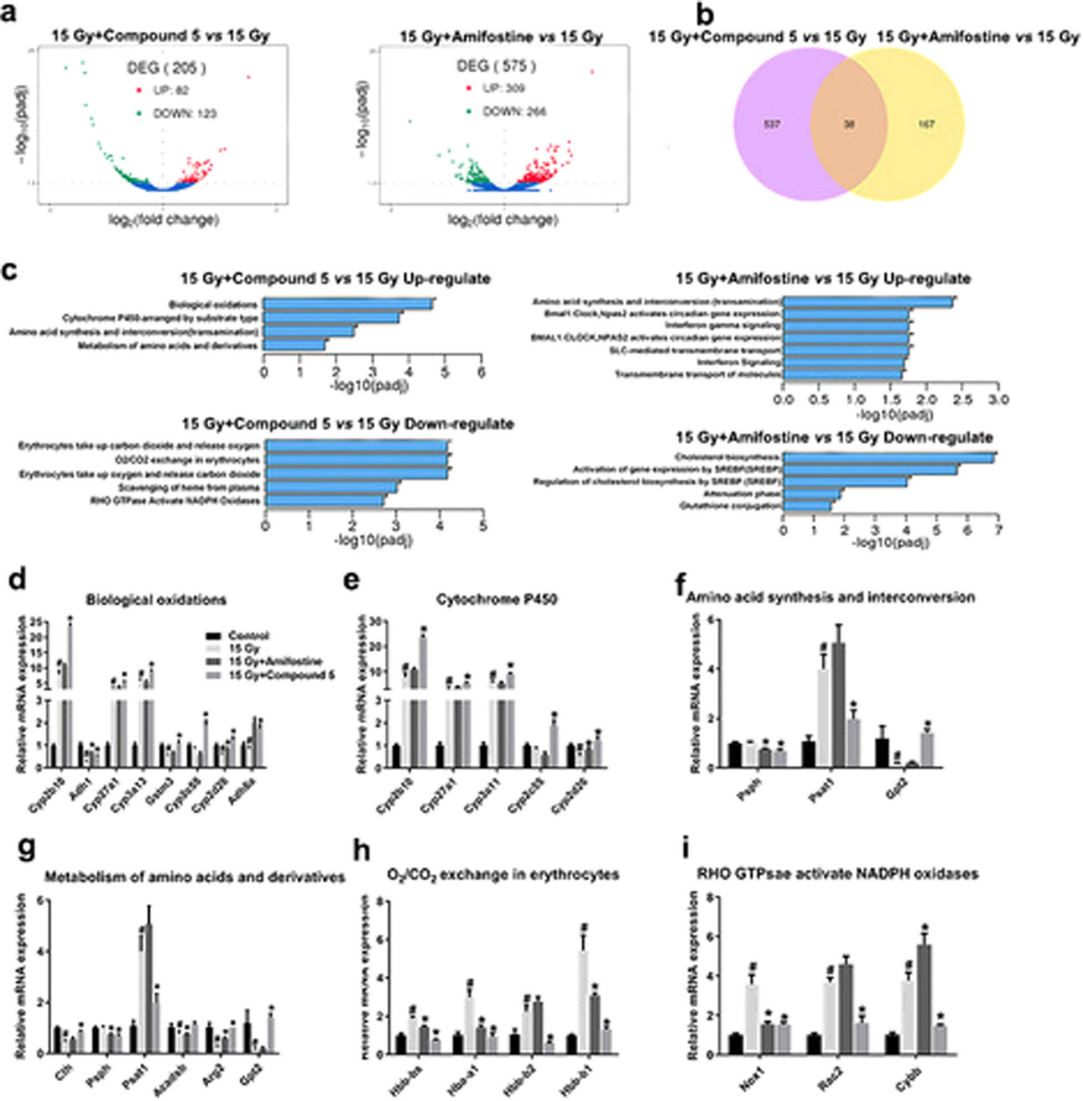
Compound 5 regulates redox balance and amino acid synthesis and metabolism pathways in irradiated mice SI. Mice were administered with NS, compound **5**, and amifostine 20 min before exposure to 15 Gy ABI as described in the “Materials and methods” section; SI crypt was obtained 4 h after ABI. **a** Volcano plots show the number of regulated genes in intestine crypt after the irradiated mice were treated with compound **5** or amifostine; **b** Venn image shows the overlap genes regulated by both compound **5** and amifostine; **c** KEGG pathway analysis shows that compound **5** and amifostine regulate the different pathways; **d**–**i** realtime PCR results show the expression of genes involved in **d** biological oxidations, **e** cytochrome P450, **f** amino synthesis and interconversion, **g** metabolism of amino acids and derivatives, **h** O_2_/CO_2_ exchange in erythrocytes, and **i** RHO GTPase active NADPH oxidases pathways; #*p* < 0.05 vs Control, *p < 0.05 vs 15 Gy, *N* = 3 in **a**–**c**, *N* = 4 in **d**–**i**.

To further explore the mechanism of compound 5, a RNA-sequence experiment was executed. As shown in Fig. 7a, b, compound **5** treatments may induce 205 genes varied including 82 upregulated and 123 downregulated genes in irradiated mice crypt. Among these genes, Cth regulate conversion of cystathione into cysteine, which is necessary for the GSH synthesis^23^; Nox1 and Rac2 are the RHO GTPases Activate NADPH Oxidases, which are dedicated to production of ROS such as superoxide^24,25^. A reactome database enrichment analysis showed that compound **5** upregulated biological oxidations, amino acid synthesis, and metabolism process in intestine crypt cells process after radiation; and downregulated erythrocytes take up oxygen and release carbon dioxide, scavenging of heme from plasma, and Rho GTPase active NADPH oxidases process in intestine crypt cells after radiation (Fig. 7c–i). These data indicate that compound **5** mainly regulatse redox balance and amino acid synthesis and metabolism to protect against radiation-induced injury.

### Compound 5 inhibited ferroptosis induced by IR and ferroptosis activator

**Fig. 8:**
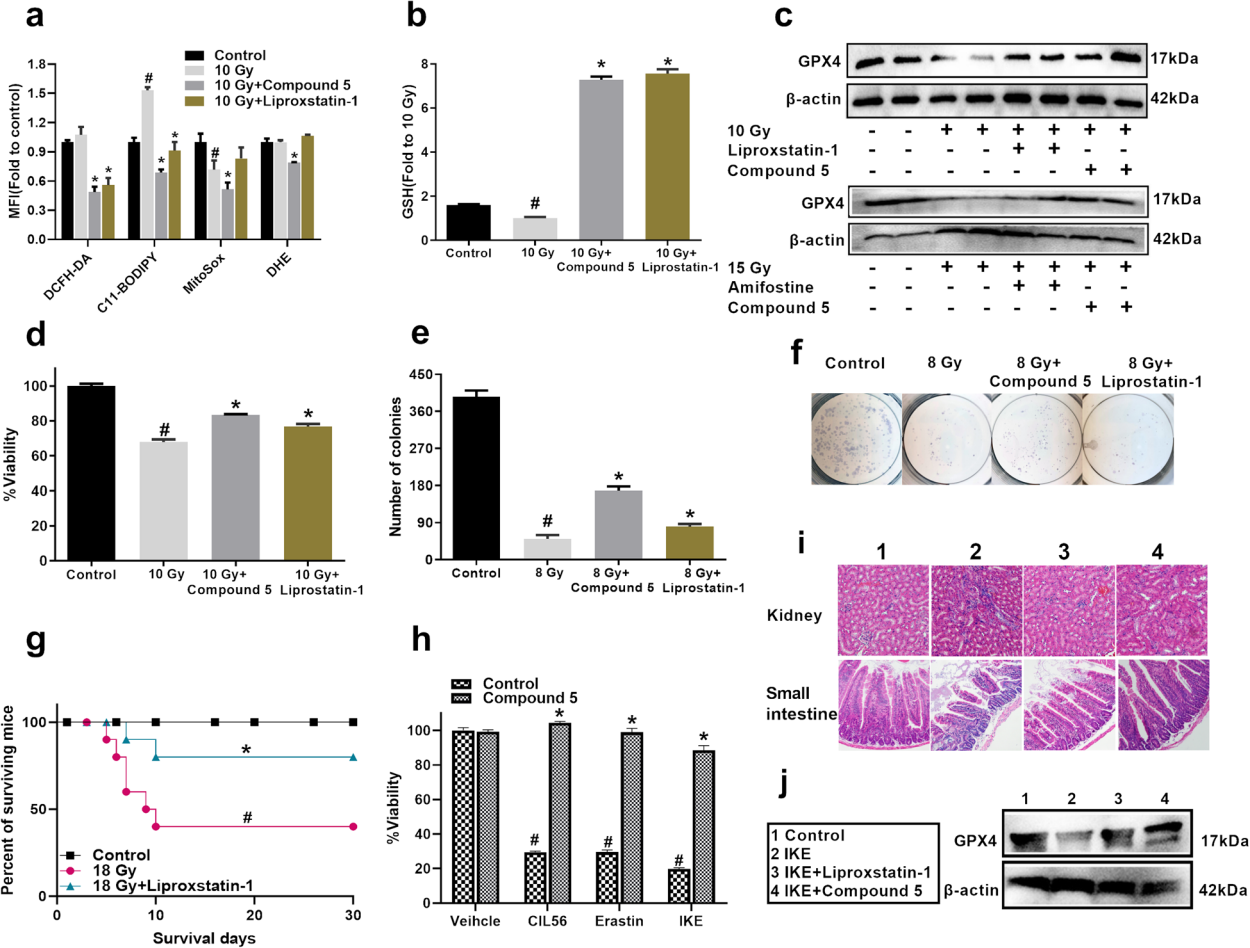
Compound 5 inhibits IR and activator-induced ferroptosis. IEC6 cells were co-cultured with 100 μM compound **5** or 50 nM liproxstatin-1 for 4 h (ROS and GSH experiments) or 24 h (survival experiment). **a** Cellular ROS level detected by DCFH-DA, C11-BODIPY, MitoSox, and DHE. **b** GSH level detected in IEC6 cells. **c** WB images showed expression of GPX4 protein in IEC6 cells (upper) and small intestine tissue (bottom). **d** Survival ratio of IEC6 cells after exposed to 10 Gy radiation. **e** Colony-forming number of IEC6 cells after exposed to 8 Gy radiation. **f** Representative images of colony forming. **g** Survival rate of mice exposed to 18 Gy ABI and treatment with 10 mg/kg liproxstatin-1 for 5 days. **h** Survival ratio of IEC6 cells after co-culture with ferroptosis activators. **i** HE images showed the morphology of small intestine and kidney after mice were administered with IKE. **j** WB images showed expression of GPX4 protein in small intestine crypts; #*p* < 0.05 vs Control, *p < 0.05 vs IR or relative ferroptosis inhibitors, *N* = 3 in **a**, *N* = 5 in **b**, *N* = 6 in **d** and **f**, *N* = 10 in **e**.

To evaluate the scavenging of compound **5** on cellular ROS level, different kinds of ROS were detected. We first detected the total ROS in irradiated IEC6 cells; as shown in Fig. 8a, there was no obvious increase in total ROS level at 4th hour after IEC6 cells were exposed to 10 Gy radiation; then we further detected the lipid ROS, mitochondrial ROS, and superoxide free radicals levels. Results showed that the radiation induced an increase in lipid ROS. It is well known that lipid ROS accumulation leads to death by ferroptosis, so we propose the hypothesis that compound **5** may inhibit the IR-induced ferroptosis^26^. We use the ferroptosis inhibitor liproxstatin-1 as the positive control in our experiment; as shown in Fig. 8a, similar to liproxstatin-1, compound **5** decreased the total ROS and lipid ROS level. Ferroptosis is characterized by the depletion of GSH, loss of activity of glutathione peroxidase 4 (GPX4), and the subsequent accumulation of lipid ROS^27,28^, so next we detected the GSH level and GPX4 protein expression. As shown in in Fig. 8b, IR induced the decrease of the GSH level; compound **5** or liproxstatin-1 treatment could increase the GSH content greatly, even exceed the GSH level in control group. Compound **5** upregulated the expression of GPX4 protein in both IEC6 cells and irradiated SI tissue (Fig. 8c).

We next detected the resource of lipid ROS; according to the result of RNA sequencing, NOX1 was significantly upregulated in irradiated mice SI crypt, and compound **5** downregulated NOX1 proteins level significantly (Supplementary Fig. 6a), which was different from amifostine, so a NOX1 inhibitor, ML171, was used in our experiment. As shown in Supplementary Fig. 6b, c, ML171 can scavenge lipid ROS specifically and elevated GSH level IEC6 cells, suggesting that NOX1 may responsible for the lipid ROS production.

As there is no research that mentioned the effect of ferroptosis in IR-induced SI injury, we aimed to explore if inhibiting ferroptosis can alleviate IR-induced SI injury. As shown in Fig. 8d–f, liproxstatin-1 elevated the survival ratio and colony number of irradiated IEC6 cells in vitro; furthermore, 10 mg/kg liproxstatin-1 can effectively increase the survival rate of lethal ABI mice (Fig. 8g). The above data suggested that IR could activate ferroptosis in SI, and compound **5** inhibited ferroptosis and alleviated IR-induced GI syndrome.

To further confirm the effect of compound **5** on ferroptosis, we co-cultured the IEC6 cells with CIL56, erastin, and IKE to trigger ferroptosis; compound **5** could inhibit ferroptosis and elevate cell survival ratio effectively (Fig. 8h). Then we administered the mice with 20 mg/kg IKE for 5 days, as shown in (Fig. 8i). In contrast to the control group, the IKE-treated group showed the destruction of the crypt and villi structure in SI and an overall greater quantity inflammatory cell infiltration in kidney effectively. Treatment with compound **5** showed a significant improvement in destroyed crypt and villi structure and inflammatory cell infiltration; IKE can also downregulate the expression of GPX4 protein, and compound **5** could upregulate the GPX4 protein level (Fig. 8j). The above data suggest that compound **5** is a potent inhibitor of ferroptosis.

## Discussion

Thiol groups and the positively charged alkylamine binding to DNA were considered as the key points for the high efficacy of amifostine, the only small molecule radio-protector approved by FDA. One of the shortcomings for amifostine is the high toxicity, which significantly limits its clinical use. One of the speculations for the high toxicity could be from the polyamine moiety. To alleviate the toxicity of amifostine, the replacement of the polyamine moiety with polycysteine amide could be one of the solutions. l-Cysteine is a kind of nutritionally semi-essential amino acid and plays an important role in human bodies, for example, it plays vital roles in redox homeostasis^29^. With this replacement, the alkyl thiol group could be retained and kept in perpendicular to the molecule backbone away from the DNA. To keep the high efficacy, more thiol groups could be added into the molecule to form the peptides. In the meantime, to make the molecule best match to DNA, the optimization of the number of thiol groups, the different stereo-configuration cysteines (both l-configuration cysteine and d-configuration cysteine), and the modification of the sidechain had been done. Overall, there are three important concepts which were systematically considered in our compound designing: firstly, the polycysteine peptide backbone to alleviate the toxicity by the replacement of the polyamine in amifostine; secondly, the number of terminal thiol segments in the amide side chain backbone is increased to elevate ROS scavenging ability; thirdly, the perpendicular, alkyl sidechain with a terminal thiol projected away from the DNA backbone to enable ROS scavenging around DNA, the same strategy as Prc-210. Based on the concepts, a series of modified polycysteine peptides had been prepared from compound **1** to compound **9** (Fig. 1a).

Compound **1** (one thiol group), compound **3** (two thiol groups), compound **5** (three thiol groups), and compound **7** (four thiol groups) are l-configuration; among them, compound **5** demonstrated the most potent radioprotection effect against TBI, lung radiation, or ABI-induced injury. After exposure to 7.2 Gy TBI, the survival rate of compound **5** was 80% (Fig. 1b, c), and the survival rates were 20%, 70%, and 0% respectively in compound **1**-, compound **3**-, and compound **7**-treated mice. l-Cysteine, with one thiol group and without any modification, gave 10% of the 30-day survival rate.

Interestingly, the corresponding d-configuration of the above compounds gave much less potency. For example, the compound **6** (three thiol groups) gave 40% of the 30-day survival rate, versus the 80% of the 30-day survival rate for compound **5**. The same as other two pair of compounds: **4** vs **3** and **1** vs **2**. The compound **4** (two thiol groups) gave 30% of the 30-day survival rate, versus the 70% of the 30-day survival rate for compound **3**. The compound **2** (one thiol groups) gave 10% of the 30-day survival rate, versus the 20% of the 30-day survival rate for compound **1**. The only difference between the pair of compounds is the l and d configuration. We believe the reason of the less potency for d-configuration compounds very likely comes from the steric confirmation mismatch binding with DNA. The l-configuration compounds sterically match better to bind with DNA to give better efficacy and the l-configuration compounds sterically mismatch to bind with DNA to give less potency.

To further clarify the correlation between the DNA binding capability and the radio-protective efficacy, each pair of the corresponding enantiomers (compounds **1**, **3**, and **5** versus compounds **2**, **4**, and **6**) had been tested for compound–DNA binding capability comparison. The calculated binding constants, respectively, are *K*_compound **1**-CT DNA_ = 7.76 × 10^3^ M^−1^; *K*_compound **3**-CT DNA_ = 4.70 × 10^3^ M^−1^; *K*_compound **5**-CT DNA_ = 9.24 × 10^3^ M^−1^; *K*_compound **2**-CT DNA_ = 4.04 × 10^3^ M^−1^; *K*_compound **4**-CT DNA_ = 2.24 × 10^3^ M^−1^; *K*_compound **6**-CT DNA_ = 1.34 × 10^3^ M^−1^. The results indicated that compound **5** showed the highest of binding capability. Also from the binding constants *K* of each of the compounds, it showed the trend: l-configuration compounds (compounds **1**, **3**, and **5**), which gave higher binding constants *K* value and therefore higher compound–DNA binding capability, exhibited more potent radiation protection efficacy than the corresponding d-configuration enantiomers (compounds **2**, **4**, and **6**), which gave relatively lower binding constants *K* value and therefore lower compound–DNA binding capability.

The only difference between each of the pair of enantiomers, which keeps the same thiol groups and same molecule weight, etc., is the compound–DNA binding capability and results in the significantly different radioprotection efficacy. This difference in the radioprotection efficacy between each of the pair of enantiomers purely comes from the compound–DNA binding capability and excludes any other possible factors. The strong correlation between the compound–DNA binding capability and the radio-protection efficacy could be established and proved clearly. Therefore, the conclusion could be made that the higher the compound–DNA binding capability, the higher the radio-protection efficacy. To our knowledge, this is the first study to prove the concept with direct comparison and clear evidence.

Compound **5** almost has the same radiation protective effect on hematopoietic system, lung and SI injury as amifostine; furthermore, their DRF was similar (1.61 and 1.71, respectively). Besides the high efficacy, compound **5** has several other desirable features, three –SH segments on the end of a perpendicular short alkyl side chain could systematically increase the ability of ROS scavenging before ROS attacking on cellular DNA and reduced intracellular DNA injury. Also, the polycysteine backbone in compound **5**, instead of the polyamine backbone in amifostine, increased the safe profile obviously. First, when the non-irradiated mice were treated with amifostine, 3746 genes were regulated including 1908 upregulated and 1308 downregulated genes; in contrast, only 865 genes changed (594 upregulated and 271 downregulated genes) in compound **5**-treated non-irradiated mice SI crypt 4 h after ABI (Supplementary Fig. 2), indicating a smaller effect of compound **5** on normal tissues than that of amifostine. Second, when the irradiated mice were treated with compound **5**, 205 genes were regulated (82 upregulated and 123 downregulated), and amifostine may induce 575 genes (309 upregulated and 266 downregulated) varied (Fig. 6b). Furthermore, the acute toxicity experiment showed that compound **5** had a larger security window, the maximum dose that allowed mice to be alive was 1000 mg/kg; the survival rate of mice after administration with 800, 1000, and 1200 mg/kg compound **5** was 100%, 100%, and 80%, respectively; in contrast, the maximum dose of amifostine that allowed mice to be alive was 500 mg/kg; the survival rate of mice after administration with 400, 500, and 600 mg/kg amifostine was 100%, 100% and 60%, respectively. The above data suggest a less toxicity of compound **5** compared to amifostine, thus breaking the defect of clinical application in which the effective dose of amifostine was very close to the toxic dose^30,31^.

To further understand the underlying mechanisms for the polycysteine as a new type of radio-protector, RNA sequencing with compound **5** and amifostine had been performed. The reactome database enrichment analysis showed that biological oxidations, amino acid synthesis, and metabolism, which are tightly linked to the regulation of ferroptosis^32^, were mainly involved in the radio-protective effect of compound **5**, while amino acid synthesis and interconversion, cholesterol biosynthesis, activation of gene expression by SREBF, and GSH conjugation pathways were mainly involved in the radio-protective effect of amifostine (Supplementary Fig. 4), so we focus on the effect of compound **5 on** IR-triggered ferroptosis. Compound **5** inhibited IR-triggered ferroptosis effectively. Lipid ROS is the main cause of ferroptosis; we first clarified that compound **5** downregulated NOX1 protein level specifically, then illustrated that NOX1 is responsible for the lipid ROS production, indicating compound **5** scavenging lipid ROS by downregulating the NOX1 protein level. Furthermore, probably compound **5** upregulated the expression of genes regulating GSH synthesis (such as Cth), increased the content of endogenous GSH, and eventually eliminated lipid ROS. We next treated the IEC6 cells with ferroptosis activator CIL56 (triggers ferroptosis upon the lipid synthetic enzyme ACC1 (ref. ^33^)), Earstin (directly binds to VDAC2/3 and inhibits the systemXc-^26^), and IKE (selectively inhibits system Xc-^34^); compound **5** treatment can elevate the above cell viability almost to the control group. In mice, compound **5** treatment ameliorated IKE-induced ferroptosis and injury in kidney and SI. The above data indicate that compound **5** inhibits ferroptosis effectively; the detailed mechanism is still undergoing investigation.

Notably, in our experiment, we observed the upregulated expression of Cyp2b10, Cyp27a1, and Cyp3a13 after mice were exposed to ABI; however, further upregulated expression of the above genes was observed when the irradiated mice were treated with compound **5**. These genes were mainly involved in biological oxidations and cytochrome P450; biological oxidations are the processes that nutrients are oxidized into water and carbon dioxide, and simultaneously produce energy; cytochrome P450 enzymes are important in the metabolism of drugs and many other types of chemicals^35^. It seems inconsistent that compound **5** can inhibit lipid ROS production, in fact, we think it is the two different process. ROS were produced by water radiolysis, compound **5** scavenged the instantaneous ROS, prevented the activation of the following ROS signaling pathway, downregulated the expression of ferroptosis-related genes and proteins, NOX1, GPX4 in our experiment. On the other hand, after compound **5** enters the organism, the cytochrome P450 enzymes may be responsible for playing the function of metabolizing compound **5**. The catalyzed reaction is accompanied by the generation of ROS and oxidative stress will further upregulate biological oxidations and cytochrome p450-related genes of Cyp2b10, Cyp27a1, and Cyp3a13. We speculate the role of compound **5** on the ROS scavenging pathway may get over the upregulation of biological oxidations, so lipid ROS was inhibited. However, it is a complicated network how compound **5** regulates the redox balance and needs to be further explored in our future work.

**Fig. 9:**
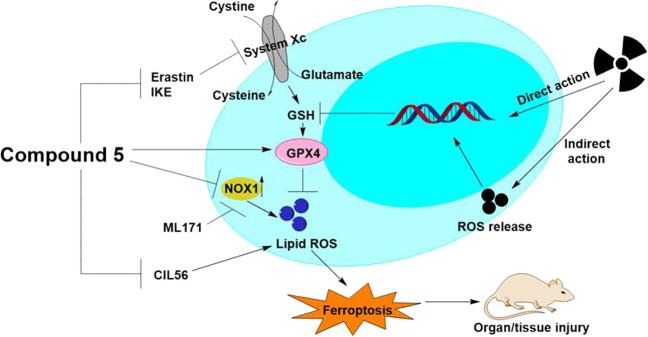
A scheme shows the radiation protective mechanism of compound 5. Ionizing radiation (IR) causes DNA double-strand breaks and oxidative damage through direct/indirect action, induces the decrease of the intracellular GSH level and down-regulation of GPX4 expression, and aggravates the effects of radiation injury by increasing the ferroptosis caused by lipid peroxidation. Compound **5** could inhibit ferroptosis pathway to play a role of radio-protection.

In summary, polycysteine, as a new type of radio-protector had been developed. Among them, compound **5** gave the highest efficacy, same as amifostine, and better safety profile. To prove the correlation between compound–DNA binding capability and the radio-protective efficacy with direct evidence, a series of l-configuration and the corresponding d-configuration polycysteine peptides had been prepared and the trend had been illustrated as the higher the binding capability, the higher the radioprotective efficacy. To elicit the mechanism of the polycysteine as radio-protector, a series of in vivo and in vitro experiments had been done; the results show that ferroptosis pathway had been involved in the radio-protection of compound **5** (Fig. 9). Therefore, in this paper, a new type of radio-protector has been designed and the detailed mechanism involved in ferroptosis had been confirmed.

## Supplementary information

Polycysteine as a new type of radio-protector ameliorated tissue injury through inhibiting ferroptosis in mice

Supplementary Table 1. Supplementary Table 2.

Supplementary Figure 1.

Supplementary Figure 2.

Supplementary Figure 3.

Supplementary Figure 4.

Supplementary Figure 5.

Supplementary Figure 6.

## References

[1] Shao L, Luo Y, Zhou D (2014). Hematopoietic stem cell injury induced by ionizing radiation. Antioxid. Redox Signal..

[2] Johnke RM, Sattler JA, Allison RR (2014). Radioprotective agents for radiation therapy: future trends. Future Oncol..

[3] Smoluk GD, Fahey RC, Ward JF (1986). Equilibrium dialysis studies of the binding of radioprotector compounds to DNA. Radiat. Res..

[4] Gauter-Fleckenstein B (2008). Comparison of two Mn porphyrin-based mimics of superoxide dismutase in pulmonary radioprotection. Free Radic. Biol. Med..

[5] Mitchell JB (1991). Inhibition of oxygen-dependent radiation-induced damage by the nitroxide superoxide dismutase mimic, tempol. Arch. Biochem. Biophys..

[6] Rabbani ZN (2007). Long-term administration of a small molecular weight catalytic metalloporphyrin antioxidant, AEOL 10150, protects lungs from radiation-induced injury. Int. J. Radiat. Oncol. Biol. Phys..

[7] Neal R, Matthews RH, Lutz P, Ercal N (2003). Antioxidant role of N-acetyl cysteine isomers following high dose irradiation. Free Radic. Biol. Med..

[8] Smoluk GD, Fahey RC, Calabro-Jones PM, Aguilera JA, Ward JF (1988). Radioprotection of cells in culture by WR-2721 and derivatives: form of the drug responsible for protection. Cancer Res..

[9] Calabro-Jones PM, Fahey RC, Smoluk GD, Ward JF (1985). Alkaline phosphatase promotes radioprotection and accumulation of WR-1065 in V79-171 cells incubated in medium containing WR-2721. Int. J. Radiat. Biol. Relat. Stud. Phys. Chem. Med..

[10] Aguilera JA, Newton GL, Fahey RC, Ward JF (1992). Thiol uptake by Chinese hamster V79 cells and aerobic radioprotection as a function of the net charge on the thiol. Radiat. Res..

[11] Zheng S, Newton GL, Ward JF, Fahey RC (1992). Aerobic radioprotection of pBR322 by thiols: effect of thiol net charge upon scavenging of hydroxyl radicals and repair of DNA radicals. Radiat. Res..

[12] Smoluk GD, Fahey RC, Ward JF (1988). Interaction of glutathione and other low-molecular-weight thiols with DNA: evidence for counterion condensation and coion depletion near DNA. Radiat. Res..

[13] Copp RR, Peebles DD, Soref CM, Fahl WE (2013). Radioprotective efficacy and toxicity of a new family of aminothiol analogs. Int. J. Radiat. Biol..

[14] Copp RR, Peebles DD, Fahl WE (2011). Synthesis and growth regulatory activity of a prototype member of a new family of aminothiol radioprotectors. Bioorg. Med. Chem. Lett..

[15] Connors K (1987). Binding Constants: The Measurement of Molecular Complex Stability.

[16] Lindemans CA (2015). Interleukin-22 promotes intestinal-stem-cell-mediated epithelial regeneration. Nature.

[17] Sato T (2009). Single Lgr5 stem cells build crypt-villus structures in vitro without a mesenchymal niche. Nature.

[18] N’Soukpoe-Kossi CN, Bourassa P, Mandeville JS, Bekale L, Tajmir-Riahi HA (2015). Structural modeling for DNA binding to antioxidants resveratrol, genistein and curcumin. J. Photochem. Photobiol. B.

[19] Weiss JF (1997). Pharmacologic approaches to protection against radiation-induced lethality and other damage. Environ. Health Perspect..

[20] Brown DQ, Graham WJ, MacKenzie LJ, Pittock JW, Shaw LM (1988). Can WR-2721 be improved upon?. Pharmacol. Ther..

[21] Komarova EA (2004). Dual effect of p53 on radiation sensitivity in vivo: p53 promotes hematopoietic injury, but protects from gastro-intestinal syndrome in mice. Oncogene.

[22] Potten CS (2004). Radiation, the ideal cytotoxic agent for studying the cell biology of tissues such as the small intestine. Radiat. Res..

[23] Lee ZW, Low YL, Huang S, Wang T, Deng LW (2014). The cystathionine gamma-lyase/hydrogen sulfide system maintains cellular glutathione status. Biochem. J..

[24] Stanley A, Thompson K, Hynes A, Brakebusch C, Quondamatteo F (2014). NADPH oxidase complex-derived reactive oxygen species, the actin cytoskeleton, and Rho GTPases in cell migration. Antioxid. Redox Signal..

[25] Miyano K, Sumimoto H (2012). Assessment of the role for Rho family GTPases in NADPH oxidase activation. Methods Mol. Biol..

[26] Xie Y (2016). Ferroptosis: process and function. Cell Death Differ..

[27] Dixon SJ, Stockwell BR (2014). The role of iron and reactive oxygen species in cell death. Nat. Chem. Biol..

[28] Yang WS, Stockwell BR (2016). Ferroptosis: death by lipid peroxidation. Trends Cell Biol..

[29] Poole LB (2015). The basics of thiols and cysteines in redox biology and chemistry. Free Radic. Biol. Med..

[30] Rose PG (1996). Amifostine cytoprotection with chemotherapy for advanced ovarian carcinoma. Semin. Oncol..

[31] Kligerman MM (1988). Final report on phase I trial of WR-2721 before protracted fractionated radiation therapy. Int. J. Radiat. Oncol. Biol. Phys..

[32] Stockwell BR (2017). Ferroptosis: a regulated cell death nexus linking metabolism, redox biology, and disease. Cell.

[33] Dixon SJ (2015). Human haploid cell genetics reveals roles for lipid metabolism genes in nonapoptotic cell death. ACS Chem. Biol..

[34] Zhang Y (2019). Imidazole ketone erastin induces ferroptosis and slows tumor growth in a mouse lymphoma model. Cell Chem. Biol..

[35] Guengerich FP, Waterman MR, Egli M (2016). Recent structural insights into cytochrome P450 function. Trends Pharmacol. Sci..

