# Supplementary material for: Polycysteine as a new type of radio-protector ameliorated tissue injury through inhibiting ferroptosis in mice
Source: Cell Death Dis. 2021 Feb 18;12(2):195. doi: 10.1038/s41419-021-03479-0 (PMC7977147; doi:10.1038/s41419-021-03479-0)
Supplement: Supplementary file 1 — Polycysteine as a new type of radio-protector ameliorated tissue injury through inhibiting ferroptosis in mice [file 41419_2021_3479_MOESM1_ESM.docx]

**Supplementary Information**

**Polycysteine as a new type of radio-protector ameliorated tissue injury through inhibiting ferroptosis in mice**

Junling Zhang^1 #^, Kui Li^1 #^, Qianru Zhang^1^, Zhimei Zhu^1^, Gongchao Huang^2^, Hongqi Tian^*1^

^1^ Tianjin Key Laboratory of Radiation Medicine and Molecular Nuclear Medicine, Institute of Radiation Medicine, Chinese Academy of Medical Science & Peking Union Medical College, Tianjin, 300000, China

^2^ KeChow Pharma, Inc., Shanghai, 201203, China

^#^Junling Zhang and Kui Li contributed equally to this work.

^*^Corresponding author, e-mail: [tianhongqi@irm-cams.ac.cn](mailto:tianhongqi@irm-cams.ac.cn)

**Experimental Section**

**Materials and Methods**

***Acute toxicity experiment***

The acute toxicity study of compound **5** was administered ip to mice at doses of 400, 500, 600, 700, 800, 900, 1000, 1100, 1200, 1600 mg/kg body weight for one time, amifostine was administrated ip to mice at doses of 400, 500, 600, 700,800 mg/kg body weight for one time, 10 mice in each group. Animals were observed for general behaviors, mortality, and food intake within 14 days. Body weight was measured twice every week.

***Index of thymus and spleen***

Body weight of individual mice was measured, Then spleen and thymus were removed and weighed. The index was calculated according to the following formula: Organ index = [organ weight (g)/body weight(g) ]× 10.

***Detection of bone marrow micronuclei***

Bone marrow cells were harvested 24h after mice were exposed to 4 Gy radiation. Femur was removed and the bone marrow cells were flushed into DMEM medium plus 20% FBS. Ten microliter cells suspension were dropped on the slide and spread as a single layer. The slide was fixed in the methanol and stained in Giemsa. Five hundred polychromatic erythrocytes were counted to evaluated the micronuclei formation.

**Results**

***Acute toxicity of* Compound 5 *by intraperitoneal injection***

No mice died when the compound **5** dose was lower than 1100mg/kg by ip injection. There were 2, 4, and 7 mice died when mice were administrated compound **5** at dose of 1100, 1200, 1600 mg/kg respectively (Supplementary Table 1). The LD50 of compound **5** is 1366 mg/kg.

**Synthetic procedures Section**

***Scheme S1.* Synthetic procedures for Compound 1**

**Synthesis of L-cysteine•HCl — N-(tert-butoxycarbonyl)-N-methyl-S-trityl-L-cysteine same as synthesis of Compound 5**

**Synthesis of tert-butyl (R)-methyl (1-(methylamino)-1-oxo-3-(tritylthio) propan-2-yl) carbamate**

N-(tert-butoxycarbonyl)-N-methyl-S-trityl-L-cysteine (5.00 g, 10.46 mmol) was dissolved in THF (25 ml) at 0-5 ℃ followed by addition of CDI (3.43 g, 21.13 mmol). The solution was stirred at 0-5 ℃ for 2h in N_2_ atmosphere, aqueous CH_3_NH_2_ solution (4.65 ml, 41.84 mmol) was added stirred at 0-5 ℃ for 2h in N_2_ atmosphere. The mixture was quenched by 2M HCl (30 ml) and extraction by DCM (100 ml). The organic phase was washed with saturated NaCl aqueous solution (100 ml) and dried with Na_2_SO_4,_ concentrated in vacuo to give crude product. The crude product was purified by silica gel chromatography (DCM/Methanol (v/v = 5/1)) to give tert-butyl (R)-methyl (1-(methylamino)-1-oxo-3-(tritylthio) propan-2-yl) carbamate as white solid (yield: 45.9%). ^1^H NMR (400MHz, CDCl_3_) *δ* 7.44-7.42 (m, 6H), 7.30-7.26 (m, 6H), 7.22-7.18 (m, 3H), 5.98 (s, 1H), 4.37 (s, 1H), 2.71-2.59 (m, 9H), 1.45 (s, 9H).

**Synthesis of (R)-3-mercapto-N-methyl-2-(methylamino) propanamide trifluoroacetate**

Tert-butyl (R)-methyl (1-(methylamino)-1-oxo-3-(tritylthio) propan-2-yl) carbamate (500.00 mg, 1.01 mmol) was dissolved in mixture solution DCM:TFA:TIPS (v/v/v=50:47:3)(20 ml) The solution was stirred at room temperature for 30min under N_2_ atmosphere, concentrated in vacuo and followed by addition of ether (10 ml) was stirred at ice bath. White solid was precipitated, filtered and washed with ether (30 ml) to give (R)-3-mercapto-N-methyl-2-(methylamino) propanamide trifluoroacetate (compound **1**) (yield: 83.0%). ^1^H NMR (400MHz, DMSO-d6) *δ* 8.92 (s, 2H), 8.53-8.52 (s, 1H), 3.90-3.87 (m, 1H), 3.01-2.91 (m, 2H), 2.68 (s, 3H), 2.53 (s, 3H). ^13^C NMR (100 MHz, DMSO) *δ* 166.59, 62.07, 31.86, 26.21, 23.82. [M+H]: Calculated: 149.0749 Found: 149.0763.

***Scheme S2.* Synthetic procedures for Compound 2**

**Synthesis of D-cysteine•HCl — N-(tert-butoxycarbonyl)-N-methyl-S-trityl-D-cysteine same as synthesis of Compound 6**

**Synthesis of tert-butyl (S)-methyl (1-(methylamino)-1-oxo-3-(tritylthio) propan-2-yl) carbamate**

N-(tert-butoxycarbonyl)-N-methyl-S-trityl-D-cysteine (3.30 g, 6.90 mmol) was dissolved in THF (20 ml) at 0-5 ℃ followed by addition of CDI (2.26 g, 13.94 mmol). The solution was stirred at 0-5 ℃ for 2h in N_2_ atmosphere, aqueous CH_3_NH_2_ solution (3.12 ml, 27.60 mmol) was added stirred at 0-5 ℃ for 2h in N_2_ atmosphere. The mixture was quenched by 2M HCl (30 ml) and extraction by DCM (100 ml). The organic phase was washed with saturated NaCl aqueous solution (100 ml) and dried with Na_2_SO_4,_ concentrated in vacuo to give crude product. The crude product was purified by silica gel chromatography (DCM/Methanol (v/v = 5/1)) to give tert-butyl (S)-methyl (1-(methylamino)-1-oxo-3-(tritylthio) propan-2-yl)carbamate as white solid (yield: 48.1%). ^1^H NMR (400MHz, CDCl_3_) *δ* 7.43-7.42 (m, 6H), 7.29-7.26 (m, 6H), 7.22-7.20 (m, 3H), 5.98 (s, 1H), 4.37 (s, 1H), 2.89-2.51 (m, 9H), 1.45 (s, 9H).

**Synthesis of (S)-3-mercapto-N-methyl-2-(methylamino) propanamide trifluoroacetate**

Tert-butyl (S)-methyl (1-(methylamino)-1-oxo-3-(tritylthio) propan-2-yl)carbamate (347.00 mg, 0.71 mmol) was dissolved in mixture solution DCM:TFA:TIPS (v/v/v=50:47:3)(15 ml) The solution was stirred at room temperature for 30min under N_2_ atmosphere, concentrated in vacuo and followed by addition of ether (10 ml) was stirred at ice bath. White solid was precipitated, filtered and washed with ether (30 ml) to give (S)-3-mercapto-N-methyl-2-(methylamino) propanamide trifluoroacetate (compound **2**) (yield: 86.0%). ^1^H NMR (400MHz, DMSO-d6) *δ* 8.92 (s, 2H), 8.53-8.52 (s, 1H), 3.89-3.87 (m, 1H), 2.97-2.91 (m, 2H), 2.70 (s, 3H), 2.53 (s, 3H). ^13^C NMR (100 MHz, DMSO) *δ* 166.60, 62.08, 31.86, 26.21, 23.83. [M+H]: Calculated: 149.0749 Found: 149.0762.

***Scheme S3.* Synthetic procedures for Compound 3**

**Synthesis of L-cysteine•HCl — (R)-2-amino-N-methyl-3-(tritylthio) propanamide same as synthesis of Compound 5**

**Synthesis of tert-butyl methyl ((R)-1-(((R)-1-(methylamino)-1-oxo-3-(tritylthio)propan-2-yl)amino)-1-oxo-3-(tritylthio)propan-2-yl)carbamate**

N-(tert-butoxycarbonyl)-N-methyl-S-trityl-L-cysteine (150.00 mg, 0.314 mmol) was dissolved in DCM (5 ml) and HOBt (63.70 mg, 0.47 mmol), EDCI (90.30 mg, 0.47 mmol) were added. The solution was stirred at room temperature for 5min and then (R)-2-amino-N-methyl-3-(tritylthio) propanamide (141.90 mg, 0.37 mmol) was added, after 30min later, the mixture was washed with saturated aqueous NaCl solution (20 ml), extracted with DCM (50 ml ×3) and dried with Na_2_SO_4,_ concentrated in vacuo and purified by silica gel chromatography (DCM/Methanol(v/v = 5/1)) to give tert-butyl methyl((R)-1-(((R)-1-(methylamino)-1-oxo-3-(tritylthio)propan-2-yl)amino)-1-oxo-3-(tritylthio)propan-2-yl)carbamate as white solid (yield: 99.2%).^1^H NMR (400MHz, CDCl_3_) *δ* 7.44-7.20(m, 30H), 6.32(s, 1H), 6.18(s, 1H), 3.99(m, 2H), 2.77-2.53(m, 10H), 1.42(s, 9H). ^13^C NMR (100 MHz, CDCl_3_) *δ* 165.20, 151.59, 139.72, 124.82, 123.29, 122.10, 62.21, 53.77, 47.67, 28.59, 27.05, 25.41, 23.60, 21.49.

**Synthesis of (R)-3-mercapto-N-((R)-3-mercapto-1-(methylamino)-1-oxopropan-2-yl)-2-(methylamino) propanamide trifluoroacetate.**

Tert-butyl methyl((R)-1-(((R)-1-(methylamino)-1-oxo-3-(tritylthio)propan-2-yl)amino)-1-oxo-3-(tritylthio)propan-2-yl)carbamate (1.10g, 1.32 mmol) was dissolved in mixture solution DCM:TFA:TIPS (v/v/v=50:47:3) (25 ml) The solution was stirred at room temperature for 5min under N_2_ atmosphere and concentrated in vacuo, then followed by addition of ether (50 ml) was stirred at ice bath. White solid was precipitated, filtered and washed with ether (200 ml) to give (R)-3-mercapto-N-((R)-3-mercapto-1-(methylamino)-1-oxopropan-2-yl)-2-(methylamino) propanamide trifluoroacetate (compound **3)** (yield: 86.9%). ^1^H NMR (400MHz, MeOD) *δ* 4.5(m, 1H), 4.09(s, 1H), 3.15-3.08(m, 2H), 2.97-2.94(m, 1H), 2.82(m, 1H), 2.77-2.74(m, 6H); 1.35(m, 2H). ^13^C NMR (100 MHz, DMSO-d_6_) *δ* 169.68, 169.52, 61.59, 56.21, 31.97, 31.12, 30.87, 26.43, 26.14, 24.0. [M+H]+: Calculated: 252.0835 Found: 252.083.

***Scheme S4.* Synthetic procedures for Compound 4**

**Synthesis of D-cysteine•HCl — (R)-2-amino-N-methyl-3-(tritylthio) propanamide same as synthesis of Compound 6**

**Synthesis of tert-butyl methyl ((S)-1-(((S)-1-(methylamino)-1-oxo-3-(tritylthio)propan-2-yl)amino)-1-oxo-3-(tritylthio)propan-2-yl)carbamate**

N-(tert-butoxycarbonyl)-N-methyl-S-trityl-D-cysteine (1.80 g, 3.77 mmol) was dissolved in DCM (30 ml) and HOBt (765.20 mg, 5.66 mmol), EDCI (1.08 g, 5.66 mmol) were added. The solution was stirred at room temperature for 5min and then (R)-2-amino-N-methyl-3-(tritylthio) propanamide (4.12 g, 3.77 mmol) was added. After 30min later, the mixture was washed with saturated aqueous NaCl solution (100 ml), then extracted with DCM (200 ml×3) and dried with Na_2_SO_4,_ concentrated in vacuo and purified by silica gel chromatography (DCM/Methanol(v/v = 5/1)) to give tert-butyl methyl((S)-1-(((S)-1-(methylamino)-1-oxo-3-(tritylthio)propan-2-yl)amino)-1-oxo-3-(tritylthio)propan-2-yl)carbamate as white solid (yield: 74.1%). ^1^H NMR (400MHz, CDCl_3_) δ 7.44-7.36(m, 15H), 7.30-7.23(m, 15H), 6.28(s, 1H), 6.15(s, 1H), 4.15-3.81(m, 3H), 2.69-2.52(m, 9H), 1.42(s, 9H). ^13^C NMR (100 MHz, CDCl_3_) δ 169.95, 144.53, 144.44, 144.35, 129.61, 129.54, 128.02, 128.00, 126.89, 126.84, 126.81, 126.78, 81.25, 58.40, 52.40, 33.53, 28.33, 28.29, 26.24, 18.45.

**Synthesis of (S)-3-mercapto-N-((S)-3-mercapto-1-(methylamino)-1-oxopropan-2-yl)-2-(methylamino) propanamide trifluoroacetate.**

Tert-butyl methyl((S)-1-(((S)-1-(methylamino)-1-oxo-3-(tritylthio)propan-2-yl)amino)-1-oxo-3-(tritylthio)propan-2-yl)carbamate (1.0 g, 1.2 mmol) was dissolved in mixture solution DCM:TFA:TIPS (v/v/v=50:47:3) (25.00 ml) The solution was stirred at room temperature for 5min under N_2_ atmosphere, concentrated *in vacuo* and then followed by addition of ether (50 ml) was stirred at ice bath. White solid was precipitated, the mixture was filtered to give (S)-3-mercapto-N-((S)-3-mercapto-1-(methylamino)-1-oxopropan-2-yl)-2-(methylamino)propanamide trifluoroacetate (compound 4) (yield: 82.3%). ^1^H NMR (400MHz, MeOD) δ 4.45(m, 1H), 4.04(m, 1H), 3.15-3.10(m, 2H), 3.05-3.00(m, 1H), 2.94-2.89(m, 1H), 2.81-2.70(m, 6H); 1.35(m, 2H). ^13^C NMR (100 MHz, DMSO) δ 170.65, 166.13, 62.22, 56.23, 31.08, 29.81, 29.55, 25.35, 24.95, 24.89, 23.68. [M+H]+: Calculated: 252.0835 Found: 252.0834.

***Scheme S5.* Synthetic procedures for Compound 5**

**Synthesis of S-trityl-L-cysteine**

L-cysteine•HCl (10.00 g, 63.45 mmol) was dissolved in DMF (120.ml) followed by addition of TrtCl (19.46 g, 69.79 mmol). The solution was heated at 60-65°C for 8h, after cooled to room temperature, 10% NaOAc (300 ml) aqueous solution was added under stirring. White solid was precipitated, the mixture was filtered and the residue was washed with water (300 ml) and acetone (200 ml), after dried (Na_2_SO_4_), S-trityl-L-cysteine was obtained (yield: 76.1%).

**Synthesis of N-(tert-butoxycarbonyl)-S-trityl-L-cysteine**

S-trityl-L-cysteine (5.00 g, 13.76 mmol) was dissolved in the mixture of dioxane (40 ml), NaOH (14 ml, 1.00 M) and water (20 ml). The solution was stirred at ice bath and Boc-anhydride was added, the mixture was stirred at room temperature for 8h and concentrated in vacuo to 20-25 ml, diluted with EtOAc ([ethyl](javascript:;) [acetate](javascript:;)) (100 ml) and adjust PH to 2-3 with NaHSO_4_ aqueous at ice bath. Then solution was extracted with EtOAc (100 ml×3). The organic phase were washed with water (100 ml) and dried with Na_2_SO_4,_ concentrated in vacuo and purified by silica gel chromatography (DCM/Methanol(v/v = 5/1)) to give N-(tert-butoxycarbonyl)-S-trityl-L-cysteine as white solid (yield: 86.2%).^1^H NMR (400MHz, CDCl_3_) *δ* 7.44-7.41(m, 6H), 7.32-7.28(m, 7H), 7.26-7.21(M, 2H), 4.92(d, 1H, *J* = 8.0 Hz), 4.13(d, 1H, *J* = 8.0 Hz), 2.70(d, 2H, *J* = 8.0 Hz), 1.46(s, 9H).

**Synthesis of N-(tert-butoxycarbonyl)-N-methyl-S-trityl-L-cysteine**

NaH (436.00 mg, 10.90 mmol) was suspended in THF (14.00 ml) and slow added of N-(tert-butoxycarbonyl)-S-trityl-L-cysteine (2.10 g, 4.53 mmol) with THF (6 ml) at ice bath followed by addition of CH_3_-I (0.93 ml, 14.95 mmol), the solution was stirred overnight at room temperature. The mixture was quenched by phosphate buffer (PH=7), saturatied aqueous NH_4_Cl solution was added to pH 2-3, and then extracted with EtOAc (50 ml). The organic phase was washed with water (50 ml) and dried with Na_2_SO_4,_ concentrated in vacuo and purified by silica gel chromatography (DCM/Methanol(v/v = 5/1)) to give N-(tert-butoxycarbonyl)-N-methyl-S-trityl-L-cysteine as white solid (yield: 60.1%).^1^H NMR (400MHz, D_2_O) *δ* 7.37-7.25(m, 15H), 3.97(s, 1H), 2.67(s, 2H), 2.61(t, 3H), 1.39(S, 4H,), 1.28(s, 5H). ^13^C NMR (100 MHz, CDCl_3_) δ 144.48, 129.58, 128.02, 126.82, 80.90, 67.00, 60.34, 59.65, 53.61, 34.10, 31.57, 30.86, 28.32.

**Synthesis of (9H-fluoren-9-yl) methyl (R)-(1-(methylamino)-1-oxo-3-(tritylthio) propan-2-yl) carbamate**

N-(((9H-fluoren-9-yl) methoxy) carbonyl)-S-trityl-L-cysteine (10.00 g, 17.07 mmol) was dissolved in THF (50 ml) at 0-5 ℃ followed by addition of CDI (5.59 g, 38.48 mmol). The solution was stirred at 0-5 ℃ for 2h in N_2_ atmosphere, aqueous CH_3_NH_2_ solution (3.03 ml, 68.28 mmol) was added stirred at 0-5 ℃ for 2h in N_2_ atmosphere. The mixture was quenched by 2M HCl (60 ml) and extraction by DCM (200 ml). The organic phase was washed with saturated NaCl aqueous solution (300 ml) and dried with Na_2_SO_4,_ concentrated in vacuo*,* methanol (20 ml) was added in crude product and stirred for overnight at room temperature. White solid was precipitated, the mixture was filtered to give (9H-fluoren-9-yl) methyl (R)-(1-(methylamino)-1-oxo-3-(tritylthio) propan-2-yl) carbamate. The filtrate was concentrated in vacuo and purified by silica gel chromatography (DCM/Methanol (v/v = 5/1)) to give (9H-fluoren-9-yl)methyl (R)-(1-(methylamino)-1-oxo-3-(tritylthio)propan-2-yl)carbamate as white solid (yield: 92.3%).^1^H NMR (400MHz, D_2_O) *δ* 7.89(d, 2H, *J* = 8.0 Hz), 7.81(d, 1H, *J* = 4.0 Hz), 7.74(d, 2H, *J* = 8.0 Hz), 7.66(d, 1H, *J* = 8.0 Hz), 7.41(t, 2H), 7.35-7.24(m, 15H), 4.32-4.20(m, 3H), 4.00(d, 1H, *J* = 8.0 Hz), 2.53(d, 3H, *J* = 4.0 Hz), 2.39(d, 2H, *J* = 8.0 Hz). ^13^C NMR (100 MHz, CDCl_3_) *δ* 170.68, 144.37, 143.64, 141.34, 129.60, 129.55, 128.10, 128.05, 127.80, 127.12, 126.95, 125.04, 120.03, 99.99, 67.40, 47.18, 33.84, 26.34.

**Synthesis of (R)-2-amino-N-methyl-3-(tritylthio) propanamide**

(9H-fluoren-9-yl) methyl (R)-(1-(methylamino)-1-oxo-3-(tritylthio) propan-2-yl) carbamate (2.00 g, 3.34 mmol) was dissolved in DMF (20 ml) followed by addition of piperidine (0.07 ml, 0.66 mmol). The solution was at stirred at room temperature for 4h. After detected and the mixture was washed with saturated aqueous NaCl solution (50 ml), then extracted with DCM (100 ml×3) and dried with Na_2_SO_4,_ concentrated in vacuo and purified by silica gel chromatography (DCM/Methanol(v/v = 5/1)) to give (R)-2-amino-N-methyl-3-(tritylthio) propanamide as slightly yellow white solid (yield: 69.7%).^1^H NMR (400MHz, D_2_O) *δ* 7.77(d, 1H, *J* = 4.0 Hz), 7.36-7.23(m, 15H), 3.08(m, 1H), 2.55(d, 3H, *J* = 8.0 Hz), 2.39-2.35(m, 1H), 2.21-2.16(m, 1H), 1.80(s, 2H). ^13^C NMR (100 MHz, CDCl_3_) *δ* 173.48, 144.65, 129.64, 127.99, 126.82, 67.02, 54.06, 37.45, 25.83.

**Synthesis of (9H-fluoren-9-yl) methyl ((R)-1-(((R)-1-(methylamino)-1-oxo-3-(tritylthio) propan-2-yl) amino)-1-oxo-3-(tritylthio) propan-2-yl) carbamate**

N-(((9H-fluoren-9-yl) methoxy) carbonyl)-S-trityl-L-cysteine (100.00 mg, 0.17 mmol) was dissolved in DCM (5 ml) and HOBt (34.50 mg, 0.25 mmol), EDCI (48.90 mg, 0.25 mmol) were added. The solution was stirred at room temperature for 5min and then (R)-2-amino-N-methyl-3-(tritylthio) propanamide (76.80 mg, 0.21 mmol) was added, After 30min later, the mixture was washed with saturated aqueous NaCl solution (20 ml), then extracted with DCM (50 ml ×3) and dried with Na_2_SO_4,_ concentrated in vacuo and purified by silica gel chromatography (DCM/Methanol(v/v = 5/1)) to give (9H-fluoren-9-yl)methyl ((R)-1-(((R)-1-(methylamino)-1-oxo-3-(tritylthio)propan-2-yl)amino)-1-oxo-3-(tritylthio)propan-2-yl)carbamate as white solid (yield: 99.6%). ^1^H NMR (400MHz, CDCl_3_) *δ* 7.76(m, 2H), 7.58(s, 2H), 7.45-7.16(m, 34H), 6.34(d, 1H, *J* = 8.0 Hz), 5.02(d, 1H, *J* = 8.0 Hz), 4.48-4.19(m. 4H), 3.84-3.78(m, 1H), 3.65(s, 3H), 2.69-2.59(m, 4H). ^13^C NMR (100 MHz, CDCl_3_) *δ* 169.48, 155.82, 144.42, 144.24, 144.05, 143.69, 143.61, 141.36, 129.55, 129.37, 128.33, 128.20, 128.11, 127.88, 127.16, 127.09, 126.99, 126.89, 125.04, 124.99, 120.10, 67.45, 67.31, 67.23, 67.11, 53.79, 52.00, 47.13, 34.13, 33.15, 26.26, 25.85.

**Synthesis of (R)-2-amino-N-((R)-1-(methylamino)-1-oxo-3-(tritylthio) propan-2-yl)-3-(tritylthio) propanamide**

(9H-fluoren-9-yl) methyl ((R)-1-(((R)-1-(methylamino)-1-oxo-3-(tritylthio) propan-2-yl) amino)-1-oxo-3-(tritylthio) propan-2-yl) carbamate (3.60 g, 3.80 mmol) was dissolved in DMF (15 ml) and piperidine (0.07 ml, 0.76 mmol) was added. The solution was stirred at room temperature for 4h, washed with saturated aqueous NaCl solution (30 ml), then extracted with DCM (50 ml×3) and dried with Na_2_SO_4,_ concentrated in vacuo and purified by silica gel chromatography (DCM/Methanol(v/v = 5/1)) to give (R)-2-amino-N-((R)-1-(methylamino)-1-oxo-3-(tritylthio)propan-2-yl)-3-(tritylthio)propanamide as white solid (yield: 47.4%). ^1^H NMR (400MHz, CDCl_3_) *δ* 7.45-7.19(m, 30H), 7.10(d, 1H, *J* = 8.0 Hz), 6.26(s, 1H), 4.02(d, 1H, *J* = 8.0 Hz), 2.82-2.69(m, 3H), 2.60(d, 3H, *J* = 4.0 Hz), 2.53-2.48(m, 2H). ^13^C NMR (100 MHz, CDCl_3_) *δ* 173.14, 170.14, 144.45, 144.44, 129.59, 129.52, 128.09, 128.03, 126.94, 126.85, 67.11, 67.05, 53.94, 52.04, 36.48, 33.21, 26.02.

**Synthesis of tert-butyl methyl((4R,7R,10R)-3,6,9-trioxo-13,13,13-triphenyl-4,7-bis((tritylthio) methyl)-12-thia-2,5,8-triazatridecan-10-yl) carbamate**

N-(tert-butoxycarbonyl)-N-methyl-S-trityl-L-cysteine (509.00 mg, 1.07 mmol) was dissolved in DCM (10 ml) and HOBt (218.00 mg, 1.61mmol), EDCI (309.00 mg, 1.61 mmol) were added. The solution was stirred at room temperature for 5min and then (R)-2-amino-N-((R)-1-(methylamino)-1-oxo-3-(tritylthio)propan-2-yl)-3-(tritylthio)propanamide (924.00 mg, 1.28 mmol) was added, after 30min later, the mixture was washed with saturated aqueous NaCl solution (30 ml), then extracted with DCM (50 ml ×3) and dried with Na_2_SO_4,_ concentrated in vacuo and purified by silica gel chromatography (DCM/Methanol(v/v = 5/1)) to give tert-butyl methyl((4R,7R,10R)-3,6,9-trioxo-13,13,13-triphenyl-4,7-bis((tritylthio)methyl)-12-thia-2,5,8-triazatridecan-10-yl)carbamate as white solid (yield: 77.9%). ^1^H NMR (400MHz, CDCl_3_) *δ* 7.45-7.12(m, 45H), 6.57(t, 2H), 5.64(s, 1H), 4.18(d, 2H,), 3.63(d, 1H), 2.84-2.28(m, 12H), 1.42(s, 9H). ^13^C NMR (100 MHz, CDCl_3_) *δ* 170.31, 144.52, 144.02, 129.60, 129.34, 128.28, 128.15, 128.07, 127.07, 126.87, 80.98, 67.00, 66.88, 60.45, 58.72, 52.51, 52.21, 33.83, 33.03, 31.64, 30.08, 28.35, 25.77, 22.71, 14.27, 14.20.

**Synthesis of (R)-3-mercapto-N-((R)-3-mercapto-1-(((R)-3-mercapto-1-(methylamino)-1-oxopropan-2-yl) amino)-1-oxopropan-2-yl)-2-(methylamino) propanamide trifluoroacetate.**

Tert-butyl methyl((4R,7R,10R)-3,6,9-trioxo-13,13,13-triphenyl-4,7-bis((tritylthio)methyl)-12-thia-2,5,8-triazatridecan-10-yl)carbamate (985.00 mg, 0.83 mmol) was dissolved in mixture solution DCM:TFA:TIPS (v/v/v=50:47:3)(15 ml) The solution was stirred at room temperature for 5min under N_2_ atmosphere, concentrated in vacuo and followed by addition of ether (45 ml) was stirred at ice bath. White solid was precipitated, filtered and washed with ether(100 ml)to give (R)-3-mercapto-N-((R)-3-mercapto-1-(((R)-3-mercapto-1-(methylamino)-1-oxopropan-2-yl)amino)-1-oxopropan-2-yl)-2-(methylamino) propanamide trifluoroacetate (compound **5)** (yield: 83.9%). ^1^H NMR (400MHz, DMSO-d6) *δ* 8.89 (d, 3H, *J* = 4.0 Hz), 8.38 (d, 1H, *J* = 8.0 Hz), 7.96 (d, 1H, *J* = 8.0 Hz), 4.53 (dd, 1H), 4.33 (dd, 1H), 4.04 (m, 1H), 3.38 (m, 1H), 2.91(m, 2H), 2.78-2.71(m, 3H), 2.61-2.56 (m, 3H), 2.50 (m, 3H), 2.29 (m, 1H), 1.28 (m, 1H), 1.29(m, 1H). ^13^C NMR (100 MHz, DMSO) *δ* 170.97, 170.41, 166.57, 62.20, 56.33, 55.92, 31.33, 29.99, 29.74, 25.93, 25.77, 25.37, 25.20, 23.96. [M+H]+: Calculated: 355.0927 Found: 355.0926.

***Scheme S6.* Synthetic procedures for Compound 6**

**Synthesis of S-trityl-D-cysteine**

D-cysteine•HCl (10.00 g, 63.45 mmol) was dissolved in DMF (120 ml) followed by addition of TrtCl (19.46 g, 69.79 mmol). The solution was heated at 60-65°C and stirred for 8h, after cooled to room temperature, 10% NaOAc (300 ml) aqueous solution was added under stirred. White solid was precipitated, the mixture was filtered and the residue was wash with water (300 ml) and acetone (200 ml), after dried (Na_2_SO_4_) to give S-trityl-D-cysteine (yield: 83.8%).

**Synthesis of N-(tert-butoxycarbonyl)-S-trityl-D-cysteine**

S-trityl-D-cysteine (14.33 g, 39.42 mmol) was dissolved in the mixture of dioxane (115 ml), NaOH (40 ml, 1M,) and water (50 ml). The solution was stirred at ice bath, after added Boc-anhydride, the mixture was stirred at room temperature for 8h and concentrated in vacuo to 20-25ml, diluted with EtOAc ([ethyl](javascript:;) [acetate](javascript:;)) (100 ml) and adjust PH to 2-3 with NaHSO_4_ aqueous at ice bath. Then solution was extracted with EtOAc (100 ml×3). The organic phase was washed with water (100 ml) and dried with Na_2_SO_4,_ concentrated in vacuo and purified by silica gel chromatography (DCM/Methanol(v/v = 5/1)) to give N-(tert-butoxycarbonyl)-S-trityl-D-cysteine as white solid (yield: 84.1%). ^1^H NMR (400MHz, CDCl_3_) *δ* 7.43(m, 6H), 7.32-7.28(m, 6H), 7.25-7.21(m, 3H), 4.97-4.95(d, 1H, *J* = 8.0 Hz), 4.19(d, 1H, *J* = 8.0 Hz), 2.69(d, 2H, *J* = 8.0 Hz), 1.46-1.41(s, 9H).

**Synthesis of N-(tert-butoxycarbonyl)-N-methyl-S-trityl-D-cysteine**

NaH (1.03 g, 25.87 mmol) was suspended in THF (30 ml) and slow added N-(tert-butoxycarbonyl)-S-trityl-D-cysteine (5.00 g, 10.78 mmol) with THF (15 ml) at ice bath followed by addition of CH_3_-I (2.22 ml, 35.57 mmol), the solution was stirred at room temperature for overnight. The mixture was quenched by phosphate buffer (PH=7), saturated aqueous NH_4_Cl solution was added to PH 2-3, and then extracted with EtOAc (100 ml). The organic phase were washed with water (150 ml) and dried with Na_2_SO_4,_ concentrated in vacuo and purified by silica gel chromatography (DCM/Methanol(v/v = 5/1)) to give N-(tert-butoxycarbonyl)-N-methyl-S-trityl-D-cysteine as white solid (yield: 75.7%). ^1^H NMR (400MHz, D_2_O) *δ* 7.35-7.26(m, 15H), 3.95(s, 1H), 2.65-2.50(m, 5H), 1.34(m, 9H). ^13^C NMR (100 MHz, CDCl_3_) *δ* 144.54, 129.59, 128.02, 126.80, 81.09, 67.00, 60.44, 34.00, 31.64, 28.36, 28.30.

**Synthesis of (9H-fluoren-9-yl) methyl (S)-(1-(methylamino)-1-oxo-3-(tritylthio) propan-2-yl) carbamate**

N-(((9H-fluoren-9-yl) methoxy) carbonyl)-S-trityl-D-cysteine (10.00 g, 17.07 mmol) was dissolved in THF (50 ml) at 0-5 ℃ followed by addition of CDI (5.59 g,38.48 mmol). The sulotion was stirred at 0-5 ℃ for 2h in N_2_ atmosphere, aqueous CH_3_NH_2_ solution (3.03 ml, 68.28 mmol) was added stirred at 0-5 ℃ for 2h in N_2_ atmosphere. The mixture was quenched by 2M HCl (60 ml) and extraction by DCM (200 ml). The organic phase was washed with saturated NaCl aqueous solution (300 ml) and dried with Na_2_SO_4,_ concentrated in vacuo*,* methanol (20 ml) was added in crude product and stirred at room temperature for overnight. White solid was precipitated, the mixture was filtered to give the product. The filtrate was concentrated in vacuo and purified by silica gel chromatography (DCM/Methanol (v/v = 5/1)) to give (9H-fluoren-9-yl)methyl (S)-(1-(methylamino)-1-oxo-3-(tritylthio)propan-2-yl)carbamate as white solid (yield: 94.0%). ^1^H NMR (400MHz, D_2_O) *δ* 7.89(d, 2H, *J* = 8.0 Hz), 7.82(d, 1H, *J* = 4.0 Hz), 7.74(d, 2H, *J* = 8.0 Hz), 7.67(d, 1H, *J* = 8.0 Hz), 7.42-7.38(m, 2H), 7.35-7.23(m, 16H), 4.32-4.18(m, 3H), 4.02-3.35(m, 1H), 2.53-2.50(m, 3H), 2.39(d, 2H, *J* = 4.0 Hz). ^13^C NMR (100 MHz, CDCl_3_) *δ* 170.57, 144.40, 143.67, 141.35, 129.62, 128.11, 127.82, 127.79, 127.13, 126.95, 125.04, 120.05, 67.38, 66.89, 47.20, 33.93, 26.32.

**Synthesis of (R)-2-amino-N-methyl-3-(tritylthio) propanamide**

(9H-fluoren-9-yl) methyl (S)-(1-(methylamino)-1-oxo-3-(tritylthio) propan-2-yl) carbamate (9.50 g, 15.86 mmol) was dissolved in DMF (95 ml) followed by addition of piperidine (0.33 ml, 3.17 mmol). The solution was stirred at room temperature for 4h.After detected and the mixture was washed with saturated aqueous NaCl solution (250 ml), then extracted with DCM (300 ml×3) and dried with Na_2_SO_4,_ concentrated in vacuo and purified by silica gel chromatography (DCM/Methanol(v/v = 5/1)) to give (R)-2-amino-N-methyl-3-(tritylthio) propanamide as slightly yellow white solid (yield: 71.7%). ^1^H NMR (400MHz, CDCl_3_) *δ* 7.48-7.45(m, 5H), 7.33-7.28(m, 6H), 7.26-7.21(m, 3H), 7.04(d, 1H, *J* = 8.0 Hz), 3.07-3.04(m, 1H), 2.80(d, 1H, *J* = 4.0 Hz), 2.77-2.74(m, 3H), 2.59-2.53(m, 1H).

**Synthesis of (9H-fluoren-9-yl) methyl ((S)-1-(((S)-1-(methylamino)-1-oxo-3-(tritylthio) propan-2-yl) amino)-1-oxo-3-(tritylthio) propan-2-yl) carbamate**

N-(((9H-fluoren-9-yl) methoxy) carbonyl)-S-trityl-D-cysteine (3.54 g, 6.05 mmol) was dissolved in DCM (30 ml) and HOBt (1.23 g, 9.07mmol), EDCI (1.74 g, 9.07 mmol) were added. The solution was stirred at room temperature for 5min and then (R)-2-amino-N-methyl-3-(tritylthio) propanamide (2.28 g, 6.05 mmol) was added. After 30min later, the mixture was washed with saturated aqueous NaCl solution (50 ml), then extracted with DCM (100 ml×3) and dried with Na_2_SO_4,_ concentrated in vacuo and purified by silica gel chromatography (DCM/Methanol(v/v = 5/1)) to give (9H-fluoren-9-yl)methyl ((S)-1-(((S)-1-(methylamino)-1-oxo-3-(tritylthio)propan-2-yl)amino)-1-oxo-3-(tritylthio)propan-2-yl)carbamate as white solid (yield: 88.9%). ^1^H NMR (400MHz, CDCl_3_) *δ* 7.80-7.14(m, 38H), 6.56(s, 1H), 5.79(d, 1H, *J* = 8.0 Hz), 5.16(d, 1H, *J* = 4.0 Hz), 4.43-4.19(m, 4H), 3.60-3.56(m, 1H), 3.13-3.08(m, 1H), 2.77-2.73(m, 1H), 2.62(d, 1H, *J* = 4.0 Hz), 2.49-2.46(m, 3H), 2.38-2.33(m, 1H). ^13^C NMR (100 MHz, CDCl_3_) *δ* 169.49, 155.83, 144.43, 144.06, 143.69, 143.62, 141.36, 129.55, 129.38, 128.33, 128.11, 127.88, 127.18, 127.16, 126.99, 120.10, 67.31, 67.24, 67.18, 53.79, 52.01, 47.13, 34.14, 33.17, 25.86.

**Synthesis of (S)-2-amino-N-((S)-1-(methylamino)-1-oxo-3-(tritylthio) propan-2-yl)-3-(tritylthio) propanamide**

(9H-fluoren-9-yl) methyl ((S)-1-(((S)-1-(methylamino)-1-oxo-3-(tritylthio) propan-2-yl) amino)-1-oxo-3-(tritylthio) propan-2-yl) carbamate (4.72 g, 5.08 mmol) was dissolved in DMF (20 ml) piperidine (0.09 ml, 1.10 mmol) was added. The solution was at stirred at room temperature for 4h, washed with saturated aqueous NaCl solution(50 ml), then extracted with DCM (100 ml×3) and dried with Na_2_SO_4,_ concentrated in vacuo and purified by silica gel chromatography (DCM/Methanol(v/v = 5/1)) to give (S)-2-amino-N-((S)-1-(methylamino)-1-oxo-3-(tritylthio)propan-2-yl)-3-(tritylthio)propanamide as white solid (yield: 91.3%). ^1^H NMR (400MHz, CDCl_3_) *δ* 7.45-7.19(m, 30H), 7.12(d, 1H), 6.26(d, 1H, *J* = 4.0 Hz), 4.02(d, 1H, *J* = 8.0 Hz), 2.83-2.68(m, 3H), 2.60(d, 3H, *J* = 4.0 Hz), 2.53-2.48(m, 2H). ^13^C NMR (100 MHz, CDCl_3_) *δ* 173.29, 170.17, 144.47, 129.60, 129.53, 128.09, 128.04, 126.94, 126.86, 67.10, 67.03, 53.99, 52.00, 36.95, 33.27, 26.04.

**Synthesis of tert-butyl methyl ((4S,7S,10S)-3,6,9-trioxo-13,13,13-triphenyl-4,7-bis((tritylthio) methyl)-12-thia-2,5,8-triazatridecan-10-yl) carbamate**

N-(tert-butoxycarbonyl)-N-methyl-S-trityl-D-cysteine (1.32 g, 2.77 mmol) was dissolved in DCM (20 ml) and HOBt (562.00 mg, 4.16 mmol), EDCI (797.00 mg, 4.16 mmol) were added. The solution was stirred at room temperature for 5min and then (S)-2-amino-N-((S)-1-(methylamino)-1-oxo-3-(tritylthio)propan-2-yl)-3-(tritylthio)propanamide (2.0 g, 2.77 mmol) was added. After 30min later, the mixture was washed with saturated aqueous NaCl solution(60 ml), then extracted with DCM (100 ml×3) and dried with Na_2_SO_4,_ concentrated in vacuo and purified by silica gel chromatography (DCM/Methanol(v/v = 5/1)) to give tert-butyl methyl((4S,7S,10S)-3,6,9-trioxo-13,13,13-triphenyl-4,7-bis((tritylthio)methyl)-12-thia-2,5,8-triazatridecan-10-yl)carbamate as white solid (yield: 81.3%). ^1^H NMR (400MHz, CDCl_3_) *δ* 7.46-7.13(m, 45H), 6.58(t, 2H), 5.61(s, 1H), 4.22-4.14(d, 2H), 3.77(s, 1H), 2.84-2.27(m, 12H), 1.41-1.29(m, 9H). ^13^C NMR (100 MHz, CDCl_3_) *δ* 144.48, 129.56, 129.54, 129.28, 128.25, 128.10, 128.02, 127.02, 126.82, 66.95, 66.83, 58.43, 53.45, 52.16, 32.99, 28.32, 18.47.

**Synthesis of (S)-3-mercapto-N-((S)-3-mercapto-1-(((S)-3-mercapto-1-(methylamino)-1-oxopropan-2-yl) amino)-1-oxopropan-2-yl)-2-(methylamino) propanamide trifluoroacetate.**

Tert-butyl methyl ((4S,7S,10S)-3,6,9-trioxo-13,13,13-triphenyl-4,7-bis((tritylthio) methyl)-12-thia-2,5,8-triazatridecan-10-yl) carbamate (1.00 g, 0.84 mmol) was dissolved in mixture solution DCM:TFA:TIPS (v/v/v=50:47:3) (15 ml). The solution was stirred at room temperature for 5min under N_2_ atmosphere, concentrated in vacuo and then followed by addition of ether (45 ml) was stirred at ice bath. White solid was precipitated, the mixture was filtered to give (S)-3-mercapto-N-((S)-3-mercapto-1-(((S)-3-mercapto-1-(methylamino)-1-oxopropan-2-yl)amino)-1-oxopropan-2-yl)-2-(methylamino) propanamide trifluoroacetate (compound **6)** (yield: 84.7%). ^1^H NMR (400MHz, MeOD) *δ* 4.56 (m, 1H), 4.43 (m, 1H), 4.05 (t, 1H), 3.29 (dd, 1H), 3.16-2.96(m, 2H), 2.90-2.76(m, 4H), 2.71 (m, 6H), 1.47-1.14(m, 2H). ^13^C NMR (100 MHz, DMSO) *δ* 170.88, 170.30, 166.32, 62.07, 56.26, 55.88, 31.07, 29.58, 25.49, 25.77, 25.13, 24.96, 23.80. [M+Na]+: Calculated: 377.0845 Found: 377.0738.

***Scheme S7.* Synthetic procedures for Compound 7**

**Synthesis of L-cysteine•HCl — (R)-2-amino-N-((R)-1-(methylamino)-1-oxo-3-(tritylthio) propan-2-yl)-3-(tritylthio) propanamide same as synthesis of Compound 5**

**Synthesis of (9H-fluoren-9-yl) methyl ((4R,7S,10R)-3,6,9-trioxo-13,13,13-triphenyl-4,7-bis((tritylthio) methyl)-12-thia-2,5,8-triazatridecan-10-yl) carbamate.**

N-(((9H-fluoren-9-yl) methoxy) carbonyl)-S-trityl-L-cysteine (1.62 g, 2.78 mmol) was dissolved in DCM (40 ml) and HOBt (563.78 mg, 4.17 mmol), EDCI (799.39 mg, 4.17 mmol) were added. The solution was stirred at room temperature for 5min and then (R)-2-amino-N-((R)-1-(methylamino)-1-oxo-3-(tritylthio) propan-2-yl)-3-(tritylthio) propanamide (R)-2-amino-N-((R)-1-(methylamino)-1-oxo-3-(tritylthio) propan-2-yl)-3-(tritylthio) propanamide (1.20 g, 2.78 mmol) was added. After 30min later, the mixture was washed with saturated aqueous NaCl solution (150 ml), then extracted with DCM (200 ml×3) and dried with Na_2_SO_4,_ concentrated in vacuo and purified by silica gel chromatography (DCM/Methanol(v/v = 5/1)) to give (9H-fluoren-9-yl)methyl ((4R,7S,10R)-3,6,9-trioxo-13,13,13-triphenyl-4,7-bis((tritylthio)methyl)-12-thia-2,5,8-triazatridecan-10-yl)carbamate as white solid (yield: 87.0%). ^1^H NMR (400MHz, CDCl_3_) *δ* 7.78(s, 2H), 7.35(m, 55H), 6.39(m, 3H), 4.87(s, 1H), 4.47(s, 1H), 4.29(s, 1H), 4.13(s, 2H), 3.90(s, 1H), 3.59(s, 1H), 2.60(s, 1H), 2.51(m, 8H). ^13^C NMR (100 MHz, CDCl_3_) *δ* 170.58, 169.72, 168.91, 156.43, 144.61, 144.09, 143.70, 143.47, 141.38, 129.62, 129.48, 129.32, 128.28, 128.25, 128.08, 127.90, 127.87, 127.18, 127.07, 126.90, 125.04, 124.99, 120.07, 67.67, 67.36, 67.25, 67.05, 54.46, 52.81, 52.57, 33.13, 25.93.

**Synthesis of (R)-2-amino-N-((S)-1-(((R)-1-(methylamino)-1-oxo-3-(tritylthio) propan-2-yl) amino)-1-oxo-3-(tritylthio) propan-2-yl)-3-(tritylthio) propanamide.**

(9H-fluoren-9-yl) methyl ((4R,7S,10R)-3,6,9-trioxo-13,13,13-triphenyl-4,7-bis((tritylthio) methyl)-12-thia-2,5,8-triazatridecan-10-yl)carbamate (2.75 g, 2.53 mmol) was dissolved in DMF (20 ml) followed by addition of piperidine (0.047 ml, 0.506 mmol). The solution was at stirred at room temperature for 4h. After detected, the mixture was washed with saturated aqueous NaCl solution (50 ml), then extracted with DCM (100 ml×3) and dried with Na_2_SO_4,_ concentrated in vacuo and purified by silica gel chromatography (DCM/Methanol(v/v = 5/1)) to give (R)-2-amino-N-((S)-1-(((R)-1-(methylamino)-1-oxo-3-(tritylthio)propan-2-yl)amino)-1-oxo-3-(tritylthio)propan-2-yl)-3-(tritylthio)propanamide as white solid (yield: 58.8%). ^1^H NMR (400MHz, CDCl_3_) *δ* 7.72(s, 1H), 7.48-7.15(m, 45H), 6.54(s, 1H), 5.92(d, 1H), 4.17(s, 1H), 3.87(s, 1H), 3.12-2.93(m, 2H), 2.68-2.31(m, 8H).

**Synthesis of tert-butyl methyl ((4R,7S,10R,13R)-3,6,9,12-tetraoxo-16,16,16-triphenyl-4,7,10-tris((tritylthio) methyl)-15-thia-2,5,8,11-tetraazahexadecan-13-yl) carbamate.**

N-(tert-butoxycarbonyl)-N-methyl-S-trityl-L-cysteine (1.90 g, 1.22 mmol) was dissolved in DCM (20 ml) and HOBt (247.42 mg, 1.83 mmol), EDCI (350.81 mg, 1.83 mmol) were added. The solution was stirred at room temperature for 5min and then (R)-2-amino-N-((S)-1-(((R)-1-(methylamino)-1-oxo-3-(tritylthio)propan-2-yl)amino)-1-oxo-3-(tritylthio)propan-2-yl)-3-(tritylthio)propanamide (1.30 g, 1.22 mmol) was added. After 30min later, the mixture was washed with saturated aqueous NaCl solution (50 ml), then extracted with DCM (100 ml×3) and dried with Na_2_SO_4,_ concentrated in vacuo and purified by silica gel chromatography (DCM/Methanol(v/v = 5/1)) to give tert-butyl methyl((4R,7S,10R,13R)-3,6,9,12-tetraoxo-16,16,16-triphenyl-4,7,10-tris((tritylthio)methyl)-15-thia-2,5,8,11-tetraazahexadecan-13-yl)carbamate as white solid (yield: 64.5%). ^1^H NMR (400MHz, CDCl_3_) *δ* 7.46-7.15(m, 60H), 6.65-6.43(m, 2H), 6.18-5.89(m, 2H), 4.25-4.03(m, 2H), 3.72(m, 1H), 3.41(s, 1H), 2.88-2.74(m, 2H), 2.63-2.50(m, 12H), 1.39(m, 9H). ^13^C NMR (100 MHz, CDCl_3_) *δ* 169.60, 167.17, 144.49, 144.13, 129.67, 129.58, 129.38, 128.24, 128.06, 127.04, 126.86, 67.39, 67.12, 61.45, 52.77, 35.13, 33.09, 32.73, 31.03, 29.70, 28.31, 26.65, 25.89.

**Synthesis of (R)-N-methyl-2-((S)-2-((R)-2-((R)-2-(methylamino)-3-(tritylthio) propanamido)-3-(tritylthio) propanamido)-3-(tritylthio) propanamido)-3-(tritylthio) propanamide trifluoroacetate.**

Tert-butyl methyl ((4R,7S,10R,13R)-3,6,9,12-tetraoxo-16,16,16-triphenyl-4,7,10-tris((tritylthio) methyl)-15-thia-2,5,8,11-tetraazahexadecan-13-yl) carbamate (1.00 g, 0.85 mmol) was dissolved in mixture solution DCM:TFA:TIPS (v/v/v=50:47:3) (20 ml) The solution was stirred at room temperature for 5min under N_2_ atmosphere, concentrated in vacuo and then followed by addition of ether (45 ml) was stirred at ice bath. White solid was precipitated, the mixture was filtered to give (R)-N-methyl-2-((S)-2-((R)-2-((R)-2-(methylamino)-3-(tritylthio) propanamido)-3-(tritylthio) propanamido)-3-(tritylthio) propanamido)-3-(tritylthio) propanamide trifluoroacetate (compound 7) (yield: 84.7%). ^1^H NMR (400MHz, DMSO-d6) *δ* 8.90(d, 3H, *J* = 8.0 Hz), 8.46(d, 1H, *J* = 8.0 Hz), 8.19(d, 1H, *J* = 8.0 Hz), 7.90(d, 1H, *J* = 4.0 Hz), 4.58-4.52(m, 1H), 4.46-4.41(m, 1H), 4.34-4.28(m, 1H), 4.06(t, 1H), 3.07(m, 1H), 2.94-2.70(m, 8H), 2.60-2.56(m, 3H), 2.50(s, 3H), 2.41(m. 1H), 1.27(m, 1H). ^13^C NMR (100 MHz, DMSO-d_6_) *δ* 170.18, 170.06, 169.96, 169.04, 169.61, 166.61, 163.52, 159.32, 119.08, 117.73, 116.09, 72.56, 65.37, 61.92, 58.39, 56.06, 55.97, 54.23, 52.77, 50.27, 49.21, 48.93, 32.01, 31.14, 30.91, 27.31, 26.52, 26.40, 26.15, 24.23. [M+H]+: Calculated: 458.1019 Found: 458.1024.

***Scheme S8.* Synthetic procedures for Compound 8**

**Synthesis of** L-cysteine•HCl **—** methyl S-trityl-N-(S-trityl-L-cysteinyl)-L-cysteinate **same as synthesis of Compound 9**

**Synthesis of S-trityl-N-(S-trityl-L-cysteinyl)-L-cysteine**

Methyl S-trityl-N-(S-trityl-L-cysteinyl)-L-cysteinate (1.60 g, 2.21 mmol) was dissolved in MeOH (60 ml) and NaOH (1M) (5.30 ml, 6.54mmol) was added alowly. The solution was stirred at room temperature, then 80% acetic acid aqueous solution was added to PH 6-7, white solid was precipited and filtration to give S-trityl-N-(S-trityl-L-cysteinyl)-L-cysteine (yield: 60.0%). ^1^H NMR (400MHz, DMSO) *δ* 8.20(s, 1H), 7.33-7.20(m, 30H), 4.80(s, 3H), 3.35-3.32(m, 1H), 3.23-3.17(m, 1H), 2.51-2.20(m, 4H).

**Synthesis of L-cysteinyl-L-cysteine trifluoroacetate**

S-trityl-N-(S-trityl-L-cysteinyl)-L-cysteine (500 mg, 0.71 mmol) was dissolved in mixture solution DCM:TFA:TIPS (v/v/v=50:47:3) (10 ml) The solution was stirred at room temperature for 10min under N_2_ atmosphere, concentrated in vacuo and then followed by addition of ether (45 ml) stirred at ice bath. White solid was precipitated, filtered and washed with ether (100 ml) to give L-cysteinyl-L-cysteine trifluoroacetate (compound **8)** (yield: 79.4%). ^1^H NMR (400MHz, DMSO) *δ* 4.63-4.67(m, 1H), 4.13-4.10(m, 1H), 3.06-3.05(m, 4H). ^13^C NMR (100 MHz, DMSO) *δ* 171.37, 167.48, 55.01, 54.21, 26.18, 26.05, 18.30. M+: Calculated: 223.0206 Found: 223.0196.

***Scheme S9.* Synthetic procedures for Compound 9**

**Synthesis of L-cysteine•HCl — S-trityl-L-cysteine same as synthesis of Compound 5**

**Synthesis of methyl S-trityl-L-cysteinate**

S-trityl-L-cysteine (7.00 g, 19.20 mmol) was dissolved in MeOH (80 ml) and H_2_SO_4_ (3.50ml) was added slowly. The solution was heated at 70-80°C and reflux overnight. The mixture was quenched by NaHCO_3_ aqueous solution, and then extracted with DCM (150 ml×3). The organic phase were dried with Na_2_SO_4,_ concentrated in vacuo and purified by silica gel chromatography (DCM/Methanol(v/v = 20/1)) to give methyl S-trityl-L-cysteinate as white solid (yield: 67.3%). ^1^H NMR (400MHz, DMSO) *δ* 7.37-7.24(m, 15H), 3.59(s, 1H), 3.21-3.18(t, 1H), 2.43-2.38(m, 1H), 2.32-2.27(m, 1H), 1.85(s, 1H).

**Synthesis of methyl N-(N-(((9H-fluoren-9-yl) methoxy) carbonyl)-S-trityl-L-cysteinyl)-S-trityl-L-cysteinate**

N-(((9H-fluoren-9-yl) methoxy) carbonyl)-S-trityl-L-cysteine (7.00 g, 11.95 mmol) was dissolved in DCM (80 ml) and HOBt (2.01 g, 14.94 mmol), EDCI (2.86 g, 14.94 mmol) were added. The solution was stirred at room temperature for 5min and then methyl S-trityl-L-cysteinate (3.76 g, 11.95 mmol) was added. After 30min later, the mixture was washed with saturated aqueous NaCl solution (200 ml), then extracted with DCM (300 ml×3) and dried with Na_2_SO_4,_ concentrated in vacuo and purified by silica gel chromatography (DCM/Methanol(v/v = 5/1)) to give methyl N-(N-(((9H-fluoren-9-yl)methoxy)carbonyl)-S-trityl-L-cysteinyl)-S-trityl-L-cysteinate as white solid (yield: 73.7%). ^1^H NMR (400MHz, DMSO) *δ* 7.74(t, 2H), 7.43-7.32(s,2H), 7.26-7.23(m,6H), 7.22-7.20(m, 6H), 7.19-7.16(m, 25H), 6.23(d, 1H, *J* = 8.0 Hz), 4.45-4.40(m, 1H), 4.37-4.30(m, 2H), 4.20-4.18(d, 2H), 3.81-3.76(m, 1H), 3.63(s, 1H), 2.68-2.57(m, 4H).

**Synthesis of methyl S-trityl-N-(S-trityl-L-cysteinyl)-L-cysteinate**

Methyl N-(N-(((9H-fluoren-9-yl) methoxy) carbonyl)-S-trityl-L-cysteinyl)-S-trityl-L-cysteinate (5.90 g, 6.24 mmol) was dissolved in DCM (75 ml) followed by addition of DBU(0.949 g, 6.24 mmol). The solution was stirred at room temperature for 30min, washed with saturated aqueous NaCl solution (200 ml), then extracted with DCM (200 ml×3), the organic phase were dried with Na_2_SO_4,_ concentrated in vacuo and purified by silica gel chromatography (DCM/Methanol(v/v = 20/1)) to give methyl S-trityl-N-(S-trityl-L-cysteinyl)-L-cysteinate as white solid (yield: 71.7%). ^1^H NMR (400MHz, D_2_O) *δ* 8.44(d, 1H, *J* = 8.0 Hz), 7.33-7.21(m, 30H), 4.15-4.12(m, 2H), 3.52(s, 3H), 2.52-2.50(m, 4H).

**Synthesis of methyl N-(N-(N-(((9H-fluoren-9-yl)methoxy) carbonyl)-S-trityl-L-cysteinyl)-S-trityl-L-cysteinyl)-S-trityl-L-cysteinate**

N-(((9H-fluoren-9-yl) methoxy) carbonyl)-S-trityl-L-cysteine (1.94 g, 3.32 mmol) was dissolved in DCM (50 ml) and HOBt (0.56 g, 4.15 mmol), EDCI (0.79 g, 4.15 mmol) were added. The solution was stirred at room temperature for 5min and then methyl S-trityl-N-(S-trityl-L-cysteinyl)-L-cysteinate (2.00 g, 2.76 mmol) was added. After 30min later, the mixture was washed with saturated aqueous NaCl solution (100 ml), then extracted with DCM (100 ml×3) and dried with Na_2_SO_4,_ concentrated in vacuo and purified by silica gel chromatography (DCM/Methanol(v/v = 20/1)) to give methyl N-(N-(N-(((9H-fluoren-9-yl)methoxy)carbonyl)-S-trityl-L-cysteinyl)-S-trityl-L-cysteinyl)-S-trityl-L-cysteinate as white solid (yield: 75.8%). ^1^H NMR (400MHz, D_2_O) *δ* 8.44(d, 1H, *J* = 8.0 Hz), 7.90-7.67(m, 6H), 7.65-7.15(m, 49H), 4.19(m, 1H), 3.99-3.96(m, 1H), 3.57(m, 1H), 3.41-3.34(m, 6H), 2.51-2.23(m, 6H).

**Synthesis of methyl S-trityl-N-(S-trityl-N-(S-trityl-L-cysteinyl)-L-cysteinyl)-L-cysteinate**

Methyl N-(N-(N-(((9H-fluoren-9-yl) methoxy) carbonyl)-S-trityl-L-cysteinyl)-S-trityl-L-cysteinyl)-S-trityl-L-cysteinate (2.97 g, 2.31 mmol) was dissolved in DCM (40 ml) followed by addition of DBU (351.42 mg, 2.31 mmol). The solution was stirred at room temperature for 30min, washed with saturated aqueous NaCl solution (100 ml), then extracted with DCM (100 ml×3) and the organic phase was dried with Na_2_SO_4,_ concentrated in vacuo and purified by silica gel chromatography (DCM/Methanol(v/v = 20/1)) to give methyl S-trityl-N-(S-trityl-N-(S-trityl-L-cysteinyl)-L-cysteinyl)-L-cysteinate as white solid (yield: 88.7%). ^1^H NMR (400MHz, D_2_O) *δ* 8.51(d, 1H, *J* = 8.0 Hz), 8.06(s, 1H), 7.33-7.17(m, 45H), 4.35(s, 1H), 4.03-3.98(m, 1H), 3.57(m, 4H), 3.13(s, 1H), 2.44-2.37(m, 2H), 2.35-2.25(m, 3H), 1.85(s, 1H).

**Synthesis of S-trityl-N-(S-trityl-N-(S-trityl-L-cysteinyl)-L-cysteinyl)-L-cysteine**

Methyl S-trityl-N-(S-trityl-N-(S-trityl-L-cysteinyl)-L-cysteinyl)-L-cysteinate (2.00 g, 1.87 mmol) was dissolved in MeOH (60 ml) and NaOH (1M) (261.80mg, 6.54mmol) was added slowly. The solution was stirred at room temperature, then 80% acetic acid aqueous solution was added to PH 6-7, white solid was precipited and filtration to give S-trityl-N-(S-trityl-N-(S-trityl-L-cysteinyl)-L-cysteinyl)-L-cysteine (yield: 61.7%). ^1^H NMR (400MHz, DMSO) *δ* 8.30(s, 1H), 8.12(s, 1H), 7.33-7.13(m, 45H), 4.36(s, 1H), 3.97-3.95(m, 1H), 3.17(s, 1H), 2.43-2.25(m, 6H).

**Synthesis of L-cysteinyl-L-cysteinyl-L-cysteine**

S-trityl-N-(S-trityl-N-(S-trityl-L-cysteinyl)-L-cysteinyl)-L-cysteine (600 mg, 0.57 mmol) was dissolved in mixture solution DCM:TFA:TIPS (v/v/v=50:47:3) (10 ml) The solution was stirred at room temperature for 10min under N_2_ atmosphere, concentrated in vacuo and followed by addition of ether (45 ml) was stirred at ice bath. White solid was precipitated, filtered and washed with ether (100 ml) to give L-cysteinyl-L-cysteinyl-L-cysteine (compound **9**) (yield: 85.4%). ^1^H NMR (400MHz, DMSO) *δ* 8.67(d, 1H, *J* = 8.0 Hz), 8.50-8.43(m, 1H), 4.57-4.53(m, 1H), 4.45-4.40(m, 1H), 3.99-3.97(m, 1H), 2.97-2.75(m, 6H). ^13^C NMR (100 MHz, DMSO-d6) *δ* 171.65, 169.69, 167.63, 55.73, 54.93, 54.33, 27.29, 26.75, 25.93. [M+NH4]+: Calculated: 327.0614 Found: 327.0684.
