# Supplementary material for: Polycysteine as a new type of radio-protector ameliorated tissue injury through inhibiting ferroptosis in mice
Source: Cell Death Dis. 2021 Feb 18;12(2):195. doi: 10.1038/s41419-021-03479-0 (PMC7977147; doi:10.1038/s41419-021-03479-0)
Supplement: Supplementary file 2 — Supplementary Table 1. Supplementary Table 2. [file 41419_2021_3479_MOESM2_ESM.docx]

**Supplementary Table 1. Acute toxicity of compound 5 and amifostine at different doses (mg/kg)**

|  | 400 | 500 | 600 | 700 | 800 | 900 | 1000 | 1100 | 1200 | 1600 |
| --- | --- | --- | --- | --- | --- | --- | --- | --- | --- | --- |
| Compound 5 | 100% | 100% | 100% | 100% | 100% | 100% | 100% | 80% | 60% | 30% |
| Amifostine | 100% | 100% | 60% | 60% | 40% | N/A | N/A | N/A | N/A | 0% |

Normal mice were treated with different dose of compound or amifostine for 1 time, then death of mice were recorded for 14 days to evaluate the acute toxicity, value in Appendix table shows the survival frequency of mice at each dose. N=10 in each group.

**Supplementary Table 2. Sequence of primers**

| **Gene name** | **Primer sequence-Forward** | **Primer sequence-Reverse** |
| --- | --- | --- |
| **Gsta4** | AGTTCTAGTGCAGCGTGCTT | TGTGGGCAGAGTGGTTTTGT |
| **Gsta2** | AGAGCTTGATGCCAGCCTTC | TCCATCAATGCAGCCACACT |
| **Gsta1** | GCAAGGAAGGCTTTCAAGATTCA | TTGCAAAATAGCCAGGATCAACA |
| **Gm3776** | TGTCCCCCAGACCAAAGAGA | TTTGCATCCATGGGAGGCTT |
| **Gss** | CGAGCGAGTGGTGACGTAT | AATCTGAGCGATTCAGGCCC |
| **Cyb2p10** | TGCATGTCTCCAAATCTCCAGG | ACTTGGGCTATTGGGAGGAAAA |
| **Cybb** | TTTGTCAAGTGCCCCAAGGT | GGCATCTTGGAACTCCTGCT |
| **ADH1** | GCAGTGGCCAAAATCGATGG | GACAGACAGACCGACACCTC |
| **Cyp27a1** | ACAGGAGGGCAAGTACCCAA | ACCTGGTCCCCTGATTCACT |
| **Cyp3a11** | AAGCATTGAGGAGGATCACACAC | GAGAGAGCTGAAACCAGGTCC |
| **Gstm3** | TGGCCCCTTCTTCATTGGTG | TGGCACTCGAGTATTGACCTT |
| **Cyp2c55** | CCCCAAGGGCACAGAGTTAG | CAAAAACAGCTCCGTGCGAG |
| **Cyp2d26** | ACGAGGCCTTCATGCCATTC | ACTTGGGGGAAGGTAGGACC |
| **Cyp3a13** | GGCCAAAACCTCTGCCTTTC | GGCTGTCGACCGTCATACAA |
| **Adh6a** | CCATGGTTGCTGCTTGGAAC | TGGGGTATGCAGTCTCTGGT |
| **Psph** | TAAGGTTCCGTTGTGCTCGC | GAAGCATCCCTCACACACGA |
| **Psat1** | CAGCCCTTAAGCCTTTCTCCT | CGGGATGACACACCGATCAA |
| **Cth** | CTGGGTGCTGATATTTGTATGT | AAACGAAGCCGACTATTGAG |
| **Gpt2** | ACAGGGCTAATCTCGGCAG | TAGGTGCAGAGTGCCATCAC |
| **Acadsb** | AGAAGACGCTTCCCAACCTG | AGGTGCAAAGGCTACTGCAT |
| **Arg2** | TAGGGTAATCCCCTCCCTGC | AGCAAGCCAGCTTCTCGAAT |
| **Oat** | GATGGCTGGCTGTGGATCAT | TAGTGGGTTTCCGCCGTATG |
| **Aldh18a1** | CGAGCAGAAGCGCAGAAATC | ACACTAATCACATTTACCCCCTG |
| **Nox1** | AAGTTTCTCTCCCGAAGGACC | AAGTTTCTCTCCCGAAGGACC |
| **Rac2** | CACGTCCTTCTCCCAACACA | AATGTCGGGGGAGCCATTTT |
| **Hbb-b1** | GAAAGGTGAACTCCGATGAA | TGATAGCAGAGGCAGAGGATAG |
| **Hbb-b2** | AGGCTCCTAGGCAATGCGAT | TACTTGTGAGCCAGGGCAGT |
| **Hba-a1** | CTGAAGCCCTGGAAAGGATGT | AGAGCCGTGGCTTACATCAAA |
| **Hbb-bs** | TGTGTTGACTCACAACCCCA | ACTTCATCGGCGTTCACCTT |
| **Sqle** | CGCAGCGGTTACTCTGGTTA | ATTCCTCCTCAAGCAAGCCC |
| **Cyp51** | CGACTTCTATCTGCCGGCTT | CAGAAGTACCATCCCGACCG |
| **Hmgcr** | AGAGAACAAGGGTTCACGCC | CCTTGGATCCCACGCGGA |
| **Fdft1** | AAGTCTAAGAGTGATACAAAGGCA | TCAGACTTCTCCACCCGTCTT |
| **Hmgcs1** | TGGCTATAAAGCTGCGGAGG | GGTGAAAGAGCCAAAGGGGA |
| **Msmo1** | CGGGGACGCCAGGAATAAAA | TTCTCCGGCAACAGTGAGTC |
| **Lss** | GTGGCAGAGAGTAGTGCTGT | CTGCCGACCCAACTCATTCT |
| **Idi1** | AGGTCGAACCCTCCTCAAGA | GAAAGAAGCGCTGGAGGTCT |
| **Mvd** | TTCCACGTCCAGTGCCATTT | CCATCACACTCGACATCCCC |
| **Elovl6** | AGTTGTACTGGTGGGTGCAG | TGGGTGGACATGGACAACTG |
| **Fasn** | CAAGTGTCCACCAACAAGCG | GGAGCGCAGGATAGACTCAC |
| **Nfya** | GCCCGATTCCCCTTTGTTCA | CTGGACAGCAGTGACACCAC |
| **GAPDH** | TGTTTCCTCGTCCCGTAGA | CAATCTCCACTTTGCCACTG |
