# Supplementary figures and images for: Polycysteine as a new type of radio-protector ameliorated tissue injury through inhibiting ferroptosis in mice
Source: Cell Death Dis. 2021 Feb 18;12(2):195. doi: 10.1038/s41419-021-03479-0 (PMC7977147; doi:10.1038/s41419-021-03479-0)

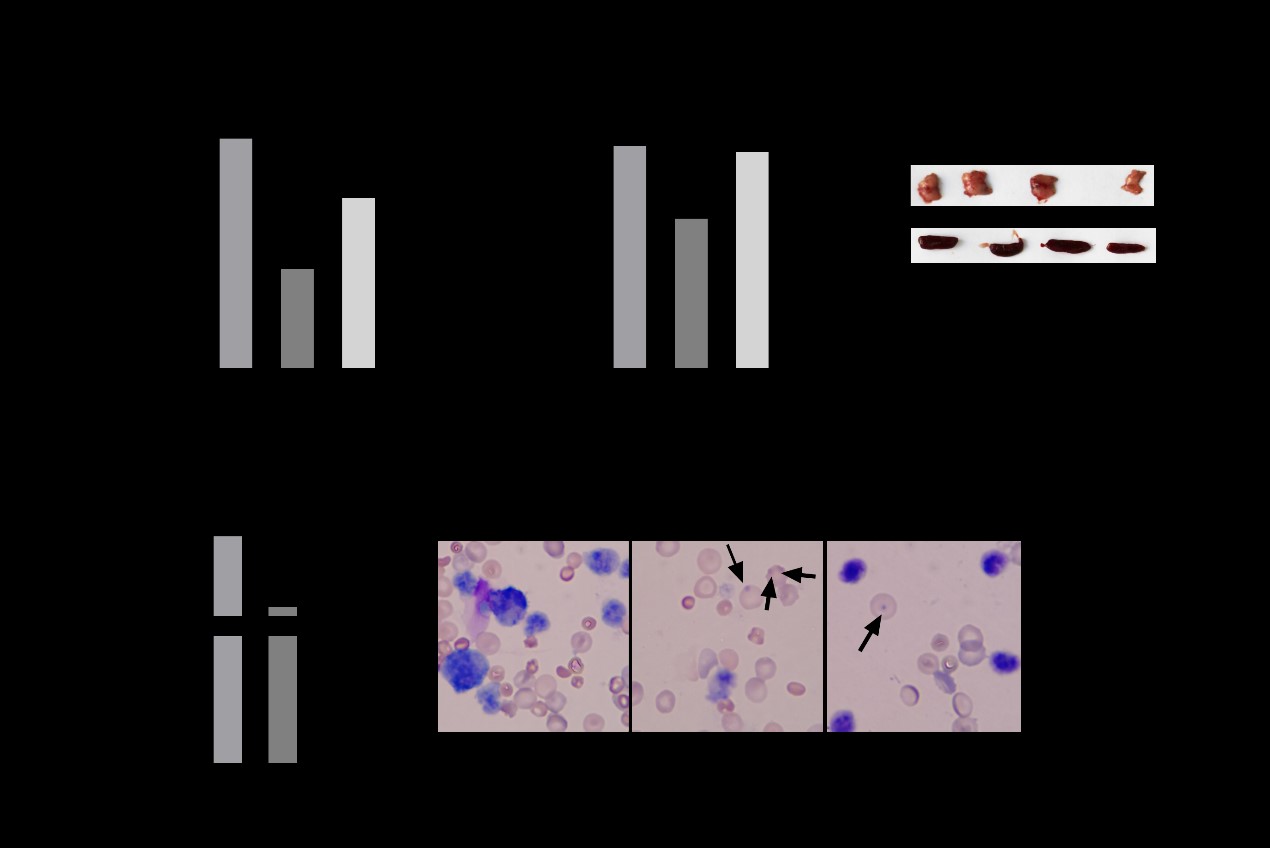

Supplement: Supplementary file 3 — Supplementary Figure 1. [file 41419_2021_3479_MOESM3_ESM.jpg]

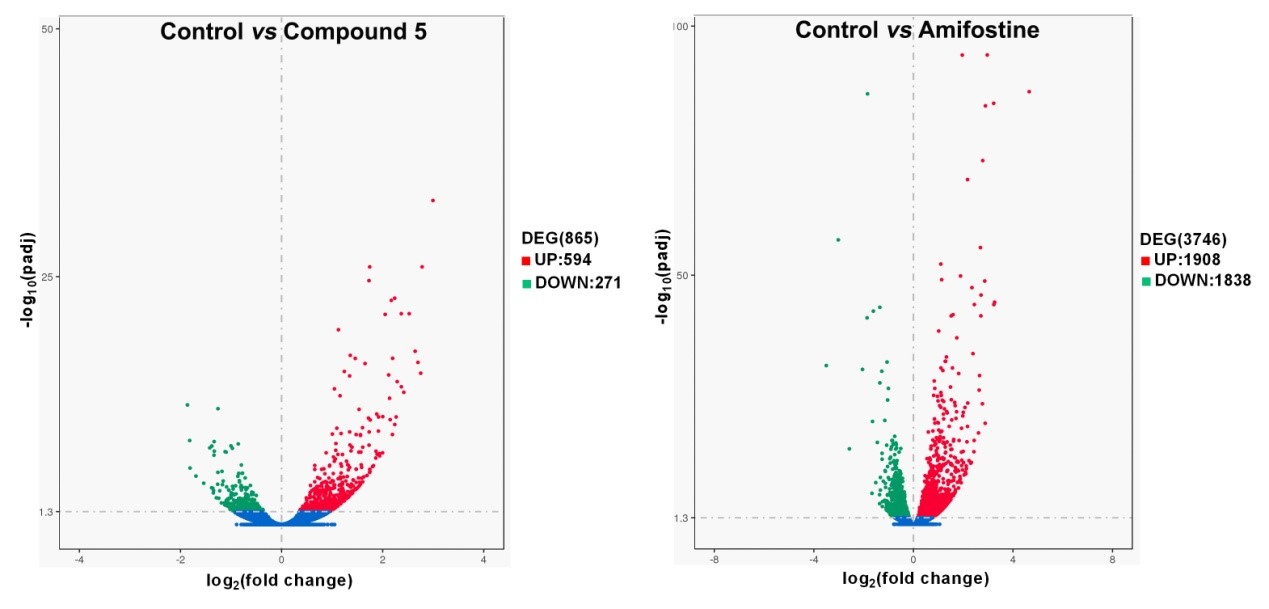

Supplement: Supplementary file 4 — Supplementary Figure 2. [file 41419_2021_3479_MOESM4_ESM.jpg]

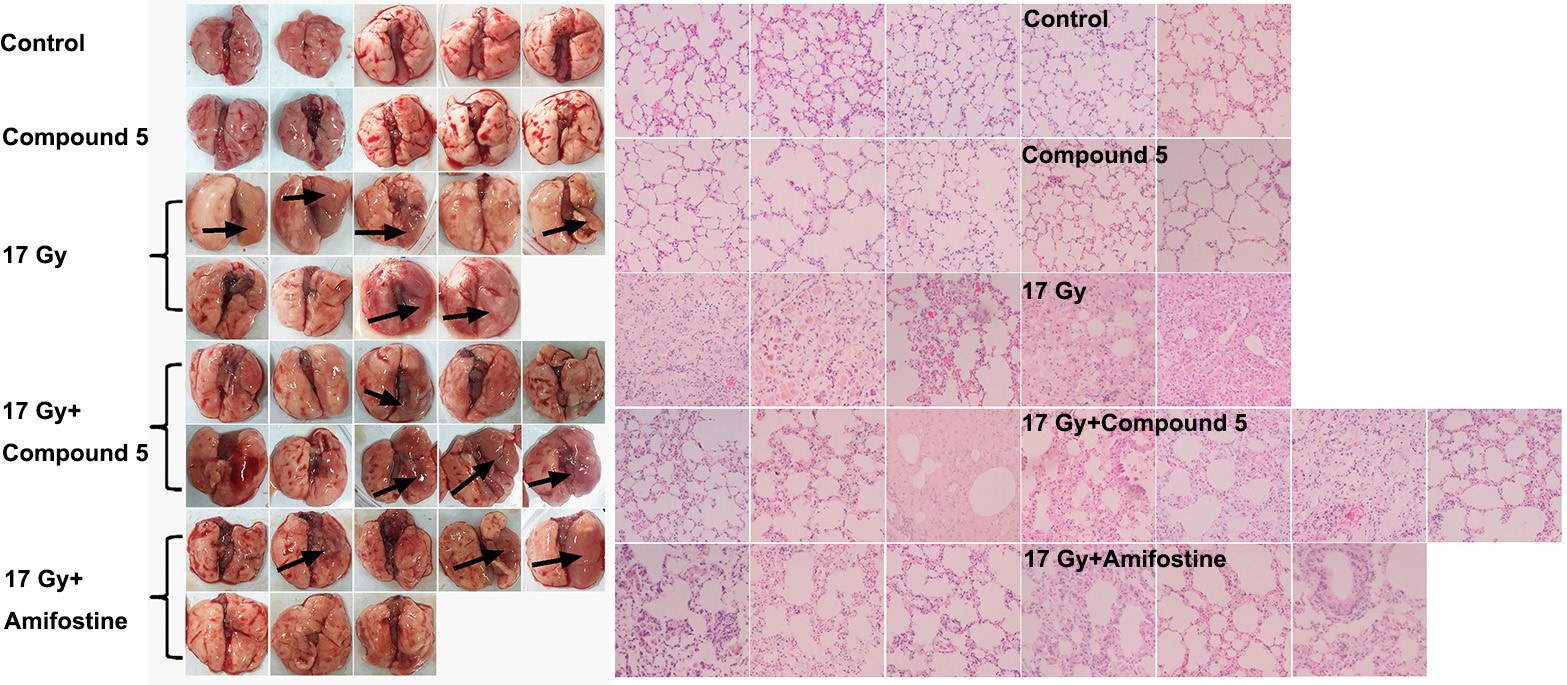

Supplement: Supplementary file 5 — Supplementary Figure 3. [file 41419_2021_3479_MOESM5_ESM.jpg]

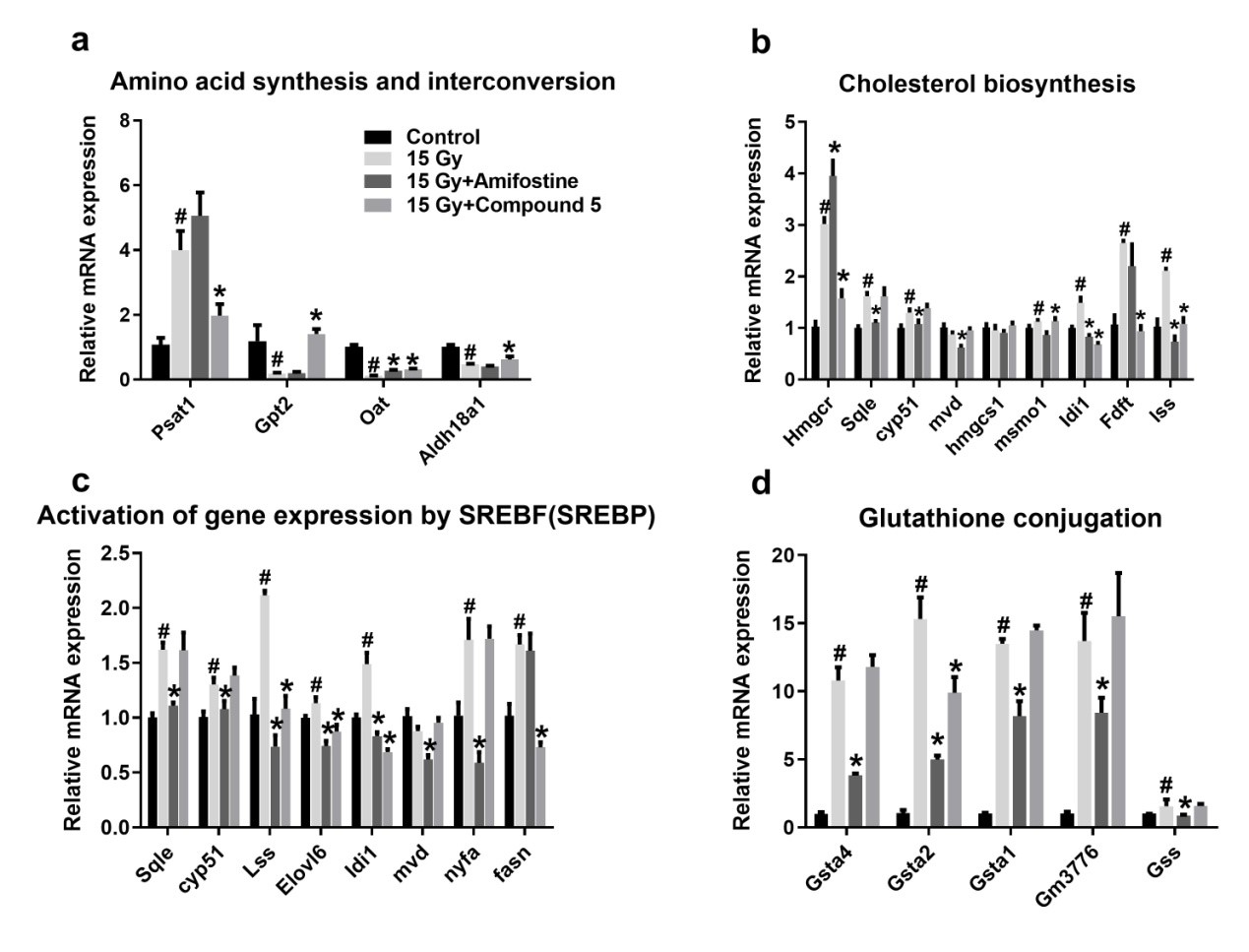

Supplement: Supplementary file 6 — Supplementary Figure 4. [file 41419_2021_3479_MOESM6_ESM.jpg]

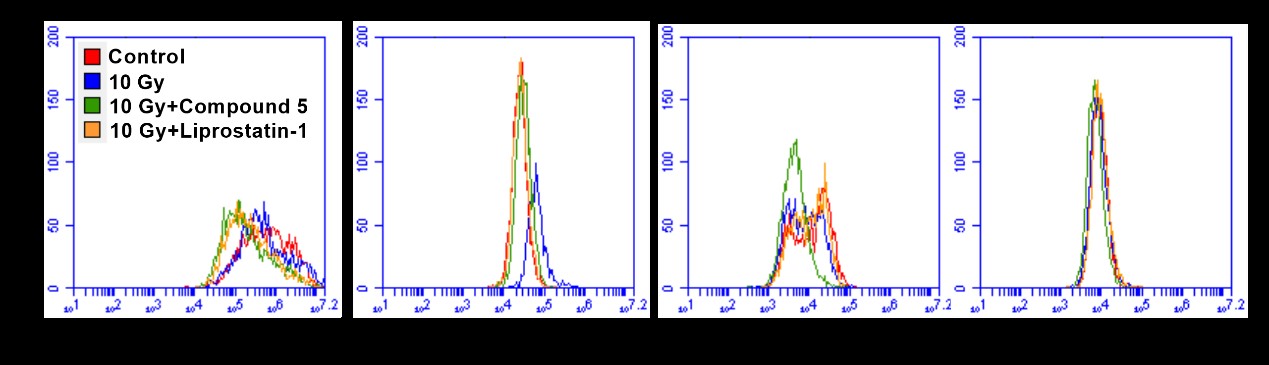

Supplement: Supplementary file 7 — Supplementary Figure 5. [file 41419_2021_3479_MOESM7_ESM.jpg]

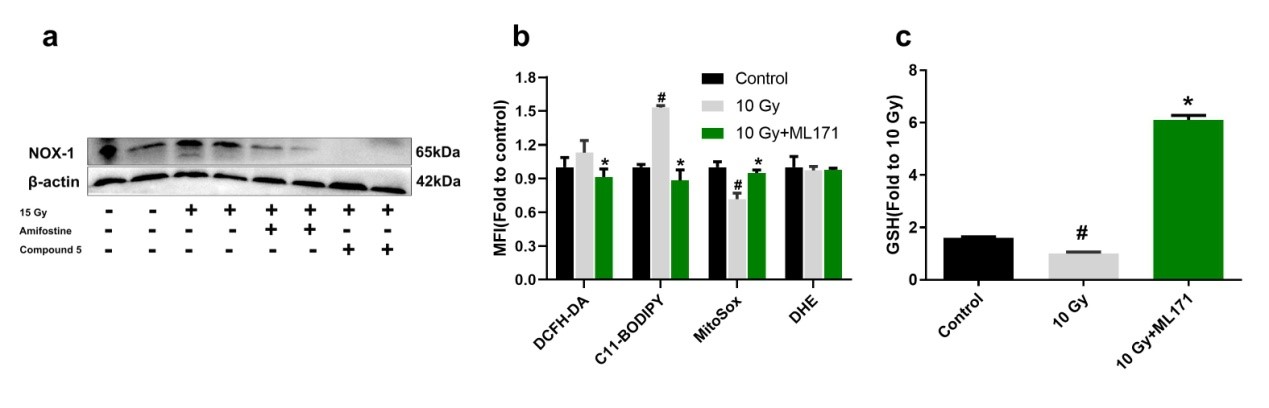

Supplement: Supplementary file 8 — Supplementary Figure 6. [file 41419_2021_3479_MOESM8_ESM.jpg]
